# Light-induced production of isobutanol and 3-methyl-1-butanol by metabolically engineered cyanobacteria

**DOI:** 10.1186/s12934-021-01732-x

**Published:** 2022-01-06

**Authors:** Shunichi Kobayashi, Shota Atsumi, Kazunori Ikebukuro, Koji Sode, Ryutaro Asano

**Affiliations:** 1 Department of Biotechnology and Life Science, Tokyo University of Agriculture and Technology, 2-24-16, Naka-cho, Koganei, Tokyo, 184-8588 Japan; 2 Department of Chemistry, University of California Davis, One Shields Avenue, Davis, CA 95616 USA; 3 Joint Department of Biomedical Engineering, The University of North Carolina at Chapel Hill and North Carolina State University, Chapel Hill, NC 27599 USA

**Keywords:** Biofuel, CcaS/CcaR two-component system, Cyanobacteria, Isobutanol, Light-regulated gene expression, Metabolic regulation, *Synechocystis* sp. PCC 6803, 3-methyl-1-butanol

## Abstract

**Background:**

Cyanobacteria are engineered via heterologous biosynthetic pathways to produce value-added chemicals via photosynthesis. Various chemicals have been successfully produced in engineered cyanobacteria. Chemical inducer-dependent promoters are used to induce the expression of target biosynthetic pathway genes. A chemical inducer is not ideal for large-scale reactions owing to its high cost; therefore, it is important to develop scaling-up methods to avoid their use. In this study, we designed a green light-inducible alcohol production system using the CcaS/CcaR green light gene expression system in the cyanobacterium *Synechocystis* sp. PCC 6803 (PCC 6803).

**Results:**

To establish the green light-inducible production of isobutanol and 3-methyl-1-butanol (3MB) in PCC 6803, keto-acid decarboxylase (*kdc*) and alcohol dehydrogenase (*adh*) were expressed under the control of the CcaS/CcaR system. Increases in the transcription level were induced by irradiation with red and green light without severe effects on host cell growth. We found that the production of isobutanol and 3MB from carbon dioxide (CO_2_) was induced under red and green light illumination and was substantially repressed under red light illumination alone. Finally, production titers of isobutanol and 3MB reached 238 mg L^−1^ and 75 mg L^−1^, respectively, in 5 days under red and green light illumination, and these values are comparable to those reported in previous studies using chemical inducers.

**Conclusion:**

A green light-induced alcohol production system was successfully integrated into cyanobacteria to produce value-added chemicals without using expensive chemical inducers. The green light-regulated production of isobutanol and 3MB from CO_2_ is eco-friendly and cost-effective. This study demonstrates that light regulation is a potential tool for producing chemicals and increases the feasibility of cyanobacterial bioprocesses.

**Graphical Abstract:**

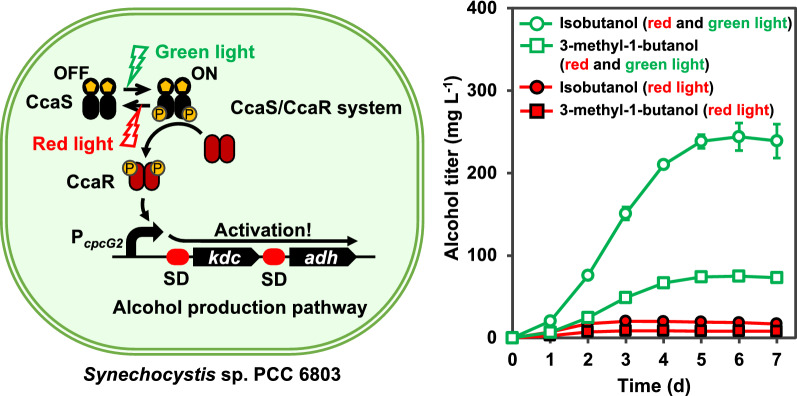

**Supplementary Information:**

The online version contains supplementary material available at 10.1186/s12934-021-01732-x.

## Background

Cyanobacteria are engineered to produce various value-added chemical compounds, such as ethanol [1, 2], isobutyraldehyde [3], isobutanol [3], 1-butanol [4], succinate [5], and 2,3-butanediol [6–8]. The expression level of a heterologous gene is one of the most critical factors in determining enzyme performance and target production, as insufficient or excessive protein levels can be detrimental to the overall yield of production [7]. The timing of protein expression during production is also of great significance, as the expression of heterologous genes during certain phases may cause metabolic imbalance or cytotoxicity [9]. Therefore, to efficiently express heterologous genes for production, an inducible promoter is often used to regulate gene expression. For most of these production systems, chemical-inducible promoters, such as an isopropyl-β-d-thiogalactopyranoside (IPTG)-inducible promoter, are used to induce the expression of target genes [10]. However, IPTG is not an ideal inducer for scale-up because of its high operational cost [11, 12]. Metal-inducible promoters are also used for this process; however, the use of such promoters restricts the efficient recycling of water, thereby increasing the burden on the environment.

Therefore, in this study, we used the CcaS/CcaR two-component system [13] to produce isobutanol and 3-methyl-1-butanol (3MB) in *Synechocystis* sp. PCC 6803 (hereafter named PCC 6803) to avoid the use of harmful chemicals and metal inducers. We have previously reported the development of light-regulated gene expression systems in cyanobacteria [14–19]. PCC 6803 harbors a green light-sensing system based on a two-component regulatory system consisting of the green light-sensing histidine kinase CcaS, its cognate response regulator CcaR, and the promoter of the *cpcG2* gene, P_*cpcG2*_ [13]. Previous studies have demonstrated that the CcaS/CcaR system strictly regulates the expression of target genes.

Several recent studies have investigated the efficiency of light-regulated gene expression systems to control metabolic pathways in microorganisms [20–23]. The application of blue light-regulated gene expression to control the isobutanol biosynthetic pathway in *Escherichia coli* and *Saccharomyces cerevisiae* leads to a similar or more efficient production of isobutanol by tuning the timing and strength of gene expression related to the biosynthetic pathway compared with production using a chemically inducible gene expression system [21, 23]. Therefore, a light-regulated gene expression system has the potential to exert more efficient control of metabolic regulation than the chemical induction used in microorganisms via easy tuning.

**Fig. 1:**
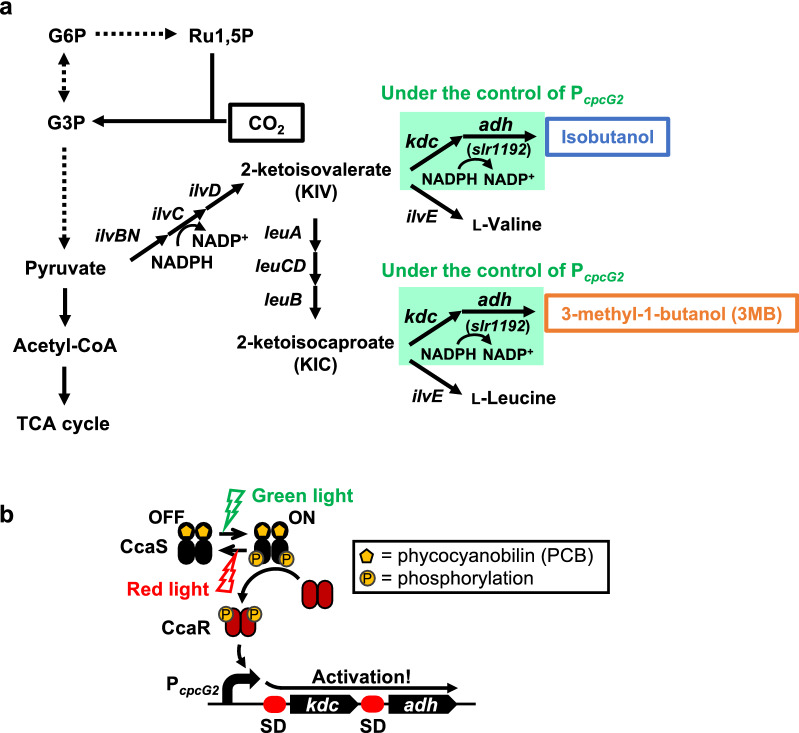
Scheme of green light-induced alcohol production in *Synechocystis* sp. PCC6803 (PCC 6803). **a** The biosynthetic pathway to produce isobutanol and 3-methyl-1-butanol (3MB) from captured CO_2_ via photosynthesis in PCC 6803. The CcaS/CcaR two-component system, which was derived from PCC6803, was applied to regulate the gene expression levels of keto-acid decarboxylase (*kdc*) and alcohol dehydrogenase (*adh*), which are required to produce isobutanol and 3MB via the amino acid biosynthesis pathway. **b** Scheme of regulation of gene expression under the control of the CcaS/CcaR system by red light and green light

Isobutanol and 3MB are valuable alcohols that serve as alternatives to gasoline and can be produced via the amino acid biosynthesis pathway in cyanobacteria. The 2-keto acid precursor, 2-ketoisovalerate, for isobutanol production is produced by the l-valine biosynthetic pathway, while the 2-keto acid precursor, 2-ketoisocaproate, for 3MB production is produced during the l-leucine biosynthetic pathway (Fig. 1a). The isobutanol biosynthetic pathway containing five genes, *alsS* (*Bacillus subtilis*) *ilvC* (*E. coli*), *ilvD* (*E. coli*), keto-acid decarboxylase (*kdc*) (*Lactococcus lactis*; hereafter named *L. lactis*), and alcohol dehydrogenase (*adh*) (*E. coli*), was introduced into *Synechococcus elongatus* PCC 7942 (hereafter named PCC 7942) [3]. The isobutanol pathway genes were expressed using an IPTG-inducible promoter. The strain was found to produce 450 mg L^−1^ of isobutanol after 6 days. Moreover, the deletion of *glgC* to shut down glycogen production further increased the production of isobutanol to 550 mg L^−1^ in 8 days [24]. The strain also produced 70 mg L^−1^ of 3MB. In addition, a high salinity stress on the engineered PCC 7942 with the isobutanol biosynthetic pathway containing the five genes further increased the isobutanol production titer to 637 mg L^−1^ in 20 days [25].

Varman et al. used a constitutive promoter to express *kdc* and *adh* in PCC 6803, resulting in the production of 90 mg L^−1^ of isobutanol in 6 days [26]. Miao et al. utilized a constitutive promoter for the isobutanol biosynthetic pathway and a KDC mutant with high catalytic activity, resulting in the production of 911 mg L^−1^ of isobutanol and 225 mg L^−1^ of 3MB in 46 days [27, 28]. Although chemical-inducible and constitutive promoters have been used to produce chemicals, light-inducible promoters will enable more controllable and economical photosynthetic production systems.

In this study, we demonstrated the green light-induced production of isobutanol and 3MB using the CcaS/CcaR two-component system in PCC 6803. The expression of the *kdc* and *adh* genes was induced by irradiation with green light under the control of P_*cpcG2*_, successfully producing isobutanol and 3MB. Furthermore, the additional expression of *ccaR* resulted in the production of 238 mg L^−1^ of isobutanol and 75 mg L^−1^ of 3MB in 5 days. This study demonstrates the potential use of the CcaS/CcaR system to produce chemicals in cyanobacteria.

## Results

### Effect of the integration of a green light-induced alcohol production system on cell growth

In cyanobacteria, isobutanol and 3MB are produced by KDC and ADH from 2-ketoisovalerate and 2-ketoisocaproate, respectively, which are 2-keto acid precursors in the l-valine and l-leucine biosynthetic pathways (Fig. 1a). To achieve green light-induced production of isobutanol and 3MB in PCC 6803, we constructed the expression vector pKT-GSS-KDC-ADH (Additional file 1: Fig. S1a), in which the CcaS/CcaR system was integrated to induce the expression of the *kdc* and *adh* genes described in the Methods section (Fig. 1b). The growth, transcription, and production of isobutanol and 3MB were then investigated by cultivating PCC 6803ΔGSS, a mutant strain without genomic CcaS/CcaR and P_*cpcG2*_ [18], with pKT-GSS-KDC-ADH, under red and green light illumination (inductive conditions: 520 nm and 660 nm, respectively; 60 µmol m^−2^ s^−1^) or only red light illumination (non-induced conditions: 660 nm, 30 µmol m^−2^ s^−1^). Irradiation with red light is necessary for photosynthesis, and we previously confirmed that it did not cause severe effects on the green light induction of gene expression from P_*cpcG2*_ [14]. Therefore, we irradiated red light during green light induction for efficient growth and alcohol production. Changes in the intensity and/or wavelength of the irradiation light may activate host metabolism, cell growth, and bioproduction. Although a twofold higher light intensity was used for irradiation, a slight delay in cell growth was observed under red and green light illumination compared to that under red light illumination (Additional file 1: Fig. S2a**)**. This delay in cell growth was also observed in the cultivation of PCC 6803ΔGSS without the vector (Additional file 1: Fig. S2b). Therefore, as reported previously, green light illumination is stressful for PCC 6803ΔGSS because of the lack of the *cpcG2* gene [18]. However, the optical density at 730 nm (OD_730_) value was maintained at approximately 70% of that under red light illumination, indicating that the stress under green light irradiation was not a serious problem in alcohol production. Compared with PCC 6803ΔGSS, an approximately 30% decrease in the OD_730_ value was observed on day 5 in PCC 6803ΔGSS with pKT-GSS-KDC-ADH (Additional file 1: Fig. S2a and b). This delay in cell growth might be due to the integration of the *kdc* and *adh* genes, which did not seriously impact the production of alcohol.

### Green light-induced production of isobutanol and 3MB

**Fig. 2:**
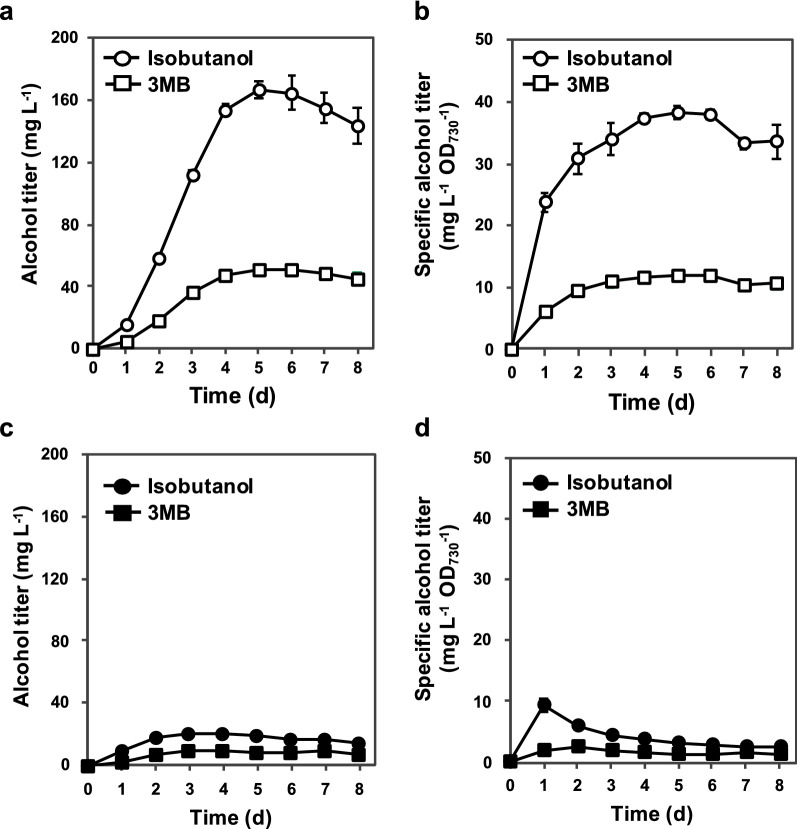
Green light-induced alcohol production using a CcaS/CcaR system. Titers and specific titers of isobutanol (circle) and 3MB (square) in the culture supernatant of *Synechocystis* sp. PCC 6803ΔGSS transformed with pKT-GSS-KDC-ADH, which was cultivated **a**, **b** under red and green light illumination or **c**, **d** under red light illumination alone. All analyses were performed in triplicate. Error bars represent the standard deviation (SD) calculated from triplicate experiments using one clone

Additional file 1: Fig. S3 shows the relative transcriptional level of the *kdc* gene in PCC 6803ΔGSS with pKT-GSS-KDC-ADH. A fivefold higher transcription level of the *kdc* gene was observed under red and green light illumination compared with that under red light illumination at 4 h. Therefore, transcription of the *kdc* gene from P_*cpcG2*_ was induced under red and green light illumination and repressed under red light illumination. Figure 2 shows the time course of alcohol production in this strain. Both isobutanol and 3MB production titers increased until day 5 under red and green light illumination, achieving 166 mg L^−1^ of isobutanol and 51 mg L^−1^ of 3MB in 5 days (Fig. 2a). The specific titers of isobutanol and 3MB on day 5 were 38 mg L^−1^ OD_730_^−1^ and 12 mg L^−1^ OD_730_^−1^, respectively (Fig. 2b). In contrast, the strain produced 19 mg L^−1^ of isobutanol and 9 mg L^−1^ of 3MB in 5 days under red light illumination alone (Fig. 2c). The specific titers of isobutanol and 3MB on day 5 were 3 mg L^−1^ OD_730_^−1^ and 1 mg L^−1^ OD_730_^−1^, respectively (Fig. 2d). We also constructed a control strain in which *kdc* and *adh* were under the control of a strong constitutive promoter, P_*trc-core*_ [29]. The isobutanol produced in the control strain was ~ 5 mg L^−1^ (~ 3 mg L^−1^ OD_730_^−1^) (Additional file 1: Fig. S4a and b), similar to a previous report using PCC 6803, the GSS-nonengineered strain expressing *kdc* and *adh* under the control of P_*trc-core*_ [29], while the production of 3MB was below the detection limit. Compared with our CcaS/CcaR system strain, the control strain exhibited slow growth (Additional file 1: Fig. S4c). Of note, the transcription of *kdc* in the control strain was observed with higher level than that in the CcaS/CcaR system strain under inductive conditions (Additional file 1: Fig. S5).

### Enhancement in the production of isobutanol and 3MB via tuning of the CcaS/CcaR system

**Fig. 3:**
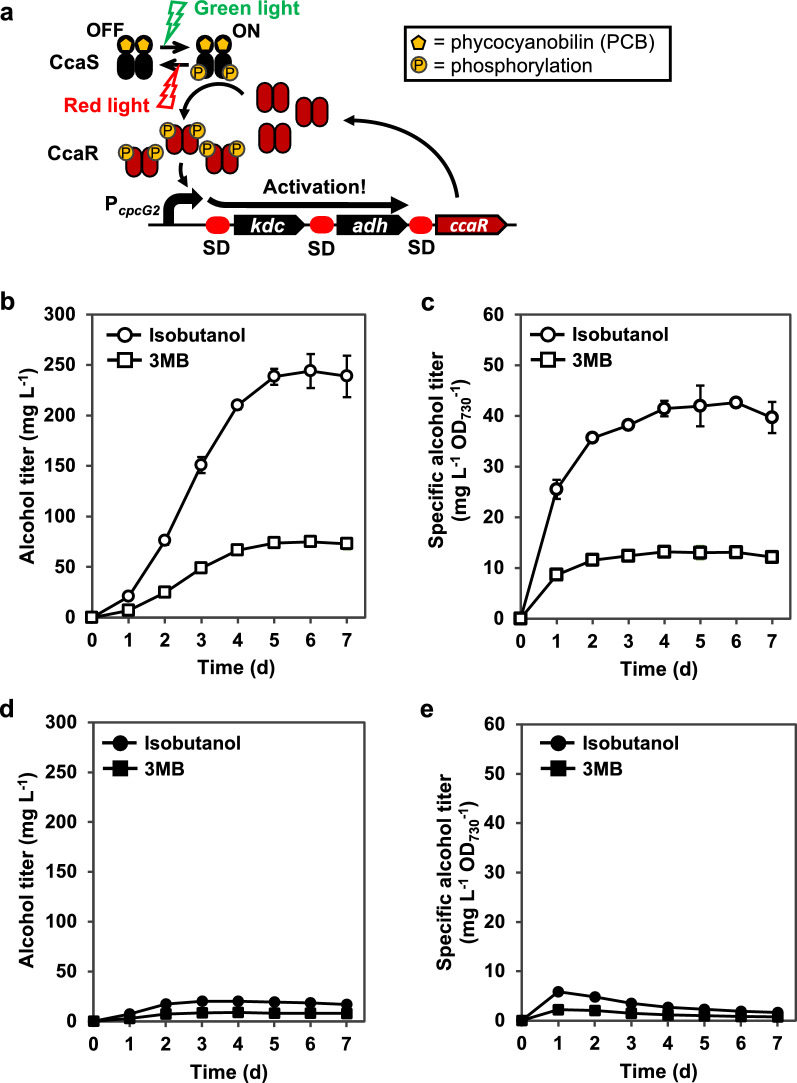
Green light-induced production of alcohol using the CcaS/CcaR system with additional expression of *ccaR*. **a** Expected scheme of gene expression regulation under the control of the CcaS/CcaR system with an additional expression of *ccaR* from P_*cpcG2*_ under green light illumination. Titers and specific titers of isobutanol (circle) and 3MB (square) by *Synechocystis* sp. PCC 6803ΔGSS transformed with pKT-GSS-KDC-ADH-CcaR, which was cultivated **b**, **c** under red and green light or **d**, **e** under red light illumination alone. All analyses were performed in triplicate. Error bars represent the standard deviation (SD) calculated from triplicate experiments using one clone

To further enhance the production of isobutanol and 3MB, we attempted to increase the expression levels of *kdc* and *adh* from P_*cpcG2*_. Our previous research revealed that the additional expression of *ccaR* using a constitutive promoter increased gene expression from P_*cpcG2*_; however, leaky expression from P_*cpcG2*_ also increased, resulting in a low ON/OFF ratio [14]. Both the high leak expression and low ON/OFF ratio negatively influenced alcohol production, as reported previously [30]. To avoid leaky expression, *ccaR* should be expressed only when the green light is illuminated. Here, we expressed an additional *ccaR* under P_*cpcG2*_ control to amplify the expression levels of *kdc* and *adh* under green light illumination (Fig. 3a). The vector pKT-GSS-KDC-ADH-CcaR was constructed by inserting an additional *ccaR* with a Shine Dalgarno (SD)-like sequence downstream of the *adh* gene in pKT-GSS-KD-ADH (Additional file 1: Fig. S1b). The PCC 6803ΔGSS strain was transformed using this vector. The resulting strain was cultivated in BG11 medium supplemented with NaHCO_3_ under red and green light illumination or red light illumination alone to investigate the level of alcohol production.

Under red and green light illumination, the strain produced 238 mg L^−1^ of isobutanol and 75 mg L^−1^ of 3MB in 5 days (Fig. 3b). The specific titers of isobutanol and 3MB on day 5 were 42 mg L^−1^ OD_730_^−1^ and 13 mg L^−1^ OD_730_^−1^, respectively (Fig. 3c). This strain produced only trace amounts of isobutanol and 3MB under red light illumination alone (Fig. 3d and e). Although the additional expression of *ccaR* improved the production of isobutanol and 3MB by 1.5-fold and 1.4-fold, respectively, the titers were not changed under non-induced conditions compared to the strain with pKT-GSS-KDC-ADH, indicating negligible leaky expression of *ccaR*.

## Discussion

The efficient production of isobutanol and 3MB in engineered cyanobacteria using a chemical inducer has been previously reported [3, 24]. In this study, we demonstrated the green light-induced production of isobutanol and 3MB without chemical inducers. A CcaS/CcaR two-component system was used to regulate the expression levels of *kdc* and *adh*. The production of isobutanol and 3MB was induced by illumination with red and green light **(**Fig. 2a and b), and both titers were successfully improved by integrating an additional *ccaR* gene (Fig. 3b and c). We have applied the CcaS/CcaR two-component system for the development of gene expression systems [14, 16, 18, 19], regulation of cell lysis [15], and cell surface display of recombinant proteins [17]. The present study highlights a new application of this system for the regulation of chemical production in cyanobacteria.

The results of isobutanol production using PCC 6803 or PCC 7942 by regulating only the *kdc* and *adh* genes are summarized in Additional file 1: Table S1. The titer and productivity achieved in this study were higher than those achieved with a chemical inducer. However, isobutanol production was improved by the chemical-inducible expression of *alsS*, *ilvC*, and *ilvD* (Additional file 1: Table S2) [3]. In contrast, the production of 3MB was comparable to that obtained with *alsS*, *ilvC*, *ilvD*, *kdc*, and *adh* (Additional file 1: Table S3). In future experiments, *alsS*, *ilvC*, and *ilvD* will be installed in the isobutanol and 3MB production strains with the CcaS/CcaR system for further improvements.

**Fig. 4:**
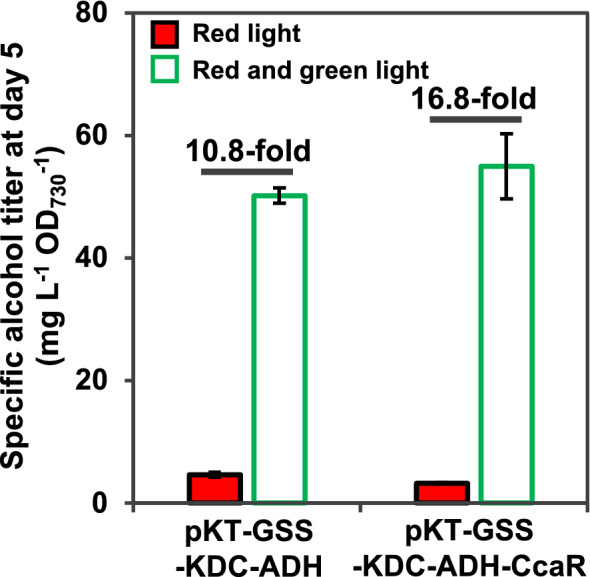
Comparison of specific alcohol titer with or without the additional expression of *ccaR*. The specific alcohol (both isobutanol and 3MB) titer in the PCC 6803ΔGSS strain transformed with pKT-GSS-KDC-ADH or pKT-GSS-KDC-ADH-CcaR at day 5, which was cultivated under red light illumination alone (red bar) or red and green light illumination (green bar). All analyses were performed in triplicate. Error bars represent the standard deviation (SD) calculated from triplicate experiments using one clone

The additional *ccaR* expression from P_*cpcG2*_ improved cell growth and the production of isobutanol and 3MB (Fig. 3bc and Additional file 1: Fig. S2c). As the specific titer under red and green light illumination did not improve with the additional expression of *ccaR* (Fig. 4), the improved cell growth increased the isobutanol and 3MB titers. Interestingly, the additional expression of *ccaR* improved the ON/OFF ratio by 1.6-fold in the specific titer of alcohols (total of isobutanol and 3MB) (p < 0.05, Student’s *t*-test) (Fig. 4), which might be caused by the strict regulation of the expression of *kdc* and *adh*. The tight repression of alcohol production with red light illumination alone may improve cell growth. The results obtained using the constitutive promoter P_*trc-core*_ also suggest the importance of strict regulation. The transcription of *kdc* gene was observed under the control of P_*trc-core*_ (Additional file 1: Fig. S5), but the production of isobutanol was only 5 mg L^−1^ and growth inhibition was observed (Additional file 1: Fig. S4a and c). This low titer may be caused by the metabolic burden due to the strong and constitutive expression of the *kdc* and *adh* genes. Furthermore, metabolic burden would also cause mutations in related genes, as observed in a previous study that produce isopropanol in PCC 7942 [30]. Even though the promoter strength of P_*cpcG2*_ was much lower than that of P_*trc-core*_ (Additional file 1: Fig. S5) [14, 19], P_*cpcG2*_ provided sufficient levels of KDC and ADH to produce isobutanol and 3MB under induced conditions without serious inhibition of growth. Therefore, this green light-induced system can be applied to the production of value-added chemicals, which require strict regulation of their own biosynthetic pathways to improve both the production titer and cell growth, such as isopropanol [30], 2,3-butandiol [6, 9], and fatty acid alcohols [31].

The intensity of green light illumination may also be important for the efficient production of alcohols in CcaS/CcaR system strain. When our strain was cultivated under various intensities of green light illumination (0 µmol m^−2^ s^−1^, 1 µmol m^−2^ s^−1^, 5 µmol m^−2^ s^−1^, 30 µmol m^−1^ s^−1^, 50 µmol m^−2^ s^−1^, or 100 µmol m^−2^ s^−1^) with red light illumination (30 µmol m^−1^ s^−1^), the transcription level of *kdc* increased in an intensity-dependent manner up to 30 µmol m^−2^ s^−1^ of green light illumination and nearly reached a plateau at higher intensities (Additional file 1: Fig. S6). These results indicate that the irradiation with 30 µmol m^−2^ s^−1^ of green light in present study is an appropriate condition but still has potential for increased productivities, while our system may also control the expression intensity, especially under the low-intensity conditions of the green light.

## Conclusions

In this study, we demonstrated that a green light-regulated system repressed the production of isobutanol and 3MB under non-induced conditions while enhancing production under induced conditions. This system also achieved better cell growth than that achieved using a constitutive promoter. Therefore, the results of this study increase the feasibility of cyanobacterial bioprocesses to produce isobutanol and 3MB from CO_2_ as an eco-friendly and cost-effective production system.

## Materials and methods

### Construction of vectors

The vector design and sequences used in this study are shown in Additional file 1: Fig. S1 and Table S4. The primer sequences used in this study are listed in Additional file 1: Table S5. All vectors were constructed using *E. coli* DH5α strain (TOYOBO Co., Ltd., Osaka, Japan). First, to apply the CcaS/CcaR system and express the isobutanol production pathway in PCC 6803, we designed the vector pKT-GSS-KDC-ADH based on pKT-GSS, which was used in our previous study for the exogenous expression of the CcaS/CcaR system in a marine cyanobacterium [16]. The green light-induced alcohol production system was constructed using two genes required for alcohol production: *kdc*, encoding keto-acid decarboxylase (KDC) derived from *L. lactis* [3], and *slr1192*, encoding alcohol dehydrogenase (ADH) derived from PCC 6803 [29]. For isobutanol production in *E. coli*, we previously prepared a synthetic *kdc* gene with codons optimized for the *E. coli* k-12 strain using the gene synthesis service of Eurofins Genomics K. K. (Tokyo, Japan). Since the codon usage between the *E. coli* k-12 strain and PCC 6803 is similar, we used this gene again in this study. The *kdc* gene was amplified via polymerase chain reaction (PCR) using a Veriti 200 (Thermo Fisher Scientific K. K., Tokyo, Japan) system from the subcloned vector using primers P01 and P02. The *slr1192* gene was also amplified via PCR from the PCC 6803 genome using primers P03 and P04 and was inserted downstream of P_*cpcG2*_ on the pKT-GSS vector with a SD-like sequence of the *cpcB* gene derived from *Synechococcus* sp. PCC 7002.

The pKT-GSS-KDC-ADH-CcaR was constructed to express additional *ccaR* under P_*cpcG2*_ control. DNA fragments encoding *kdc* and *slr1192* were amplified via PCR using pKT-GSS-KDC-ADH and primers P05 and P06. DNA fragments encoding *ccaR* with the SD-like sequence were also amplified via PCR from pKT-GSS using primers P07 and P08. These two DNA fragments were inserted downstream of P_*cpcG2*_ on pKT-GSS.

For the constitutive expression of *kdc* and *adh* in PCC 6803, a pKT-P_*trc-core*_-KDC-ADH was constructed. The gene fragments with *kdc* and *adh* were amplified via PCR from pKT-GSS-KDC-ADH using primers P09 and P10. Simultaneously, the P_*trc-core*_ [29] and terminator sequence were attached upstream of *kdc* and downstream of the *adh* gene, respectively. The DNA fragment was then inserted into the *Pst*I site of the pKT-230 vector.

### Transformation of cyanobacteria

Cyanobacterial transformation was performed as described previously [18]. In this study, the PCC 6803ΔGSS strain, the deletion mutant of the whole GSS cassette (*ccaS*, *ccaR*, P_*cpcG2*_, and *cpcG2*) we previously constructed for the engineering of *ccaS* [18], was used as the host for all experiments because the mere defect of P_*cpcG2*_ is difficult because of a partial overlap of the expression cassette of *ccaR* with the P_*cpcG2*_ in the genome of PCC 6803. To express *kdc* and *adh* under the control of the CcaS/CcaR system, we used the pKT-GSS vector, which was also constructed for the exogenous expression of the CcaS/CcaR system in a previous study [16]. The cells were transformed by electroporation using a Gene Pulser Xcell™ (Bio-Rad Laboratories, Hercules, USA), and recovered in BG11 medium at 30 °C for 1 day under red light illumination (660 nm, 30 µmol m^−2^ s^−1^), which was supplied using an LED irradiation unit (Nippon Medical & Chemical Instruments Co., Ltd., Osaka, Japan). The recovered cells were plated on BG11 agar plates supplemented with 20 µg mL^−1^ chloramphenicol and 50 µg mL^−1^ streptomycin. The agar plates were then incubated at 30 °C for 1 week under red light illumination. A single colony was inoculated into 1 mL of BG11 medium supplemented with antibiotics and incubated at 30 °C under red light illumination. The transformation was verified by PCR.

### Transcriptional analysis

Since the *kdc* and *adh* genes were expressed from the same mRNA, transcription analyses of the *kdc* gene were performed as follows to confirm the regulation of gene transcription under the control of P_*cpcG2*_. Throughout the cultivation, red light illumination (660 nm, 30 µmol m^−2^ s^−1^) and green light illumination (520 nm, 30 µmol m^−2^ s^−1^) were supplied from the bottom of the flasks using an LED irradiation unit (Nippon Medical & Chemical Instruments Co., Ltd., Osaka, Japan). The transformants of PCC 6803ΔGSS were inoculated into 20 mL fresh BG11 medium supplemented with 20 µg mL^−1^ chloramphenicol and 50 µg mL^−1^ streptomycin, at OD_730_ = 0.2, in Erlenmeyer flasks and grown at 30 °C with shaking at 100 rpm under red light illumination alone for 24 h. Then, half of the flasks were incubated under continuous red light illumination alone and the other half under both red and green light illumination (660 and 520 nm, respectively; 60 µmol m^−2^ s^−1^). Cells were harvested from 1 mL of culture medium at 0 and 4 h and immediately frozen in liquid nitrogen. Then, cDNA was synthesized using total RNA, and the transcription level of each gene was quantified as previously described [18]. Primer pairs, P11-P12 and P13-P14, were used to detect the expression of the *kdc* gene and 16S ribosomal RNA of PCC 6803, respectively. The transcription level of *kdc* was calculated using the ΔΔCt method [32], using the transcription level of 16S ribosomal RNA for normalization.

To compare the transcription level of the *kdc* gene between the P_*cpcG2*_ and P_*trc-core*_, each transformant of PCC 6803ΔGSS was inoculated as described above in Erlenmeyer flasks and cultivated under the red and green light illumination for 24 h. RNA isolation, cDNA synthesis and quantification of transcription levels were conducted as described above.

To compare the transcription level of *kdc* gene from the P_*cpcG2*_ under the different intensities of green light illumination, the PCC 6803ΔGSS strain transformed with pKT-GSS-KDC-ADH was inoculated as described above in Erlenmeyer flasks and cultivated under 0 µmol m^−2^ s^−1^, 1 µmol m^−2^ s^−1^, 5 µmol m^−2^ s^−1^, 30 µmol m^−1^ s^−1^, 50 µmol m^−2^ s^−1^, or 100 µmol m^−2^ s^−1^ of green light illumination with red light illumination (30 µmol m^−1^ s^−1^) for 24 h. RNA isolation, cDNA synthesis and quantification of transcription levels were conducted as described above.

### Evaluation of alcohol production by PCC 6803ΔGSS transformants

The production of isobutanol and 3MB by the PCC 6803ΔGSS transformants was evaluated using the culture supernatant via gas chromatography. Transformants were first inoculated into 20 mL of fresh BG11 medium supplemented with 25 mM sodium bicarbonate (NaHCO_3_), 20 µg mL^−1^ chloramphenicol, and 50 µg mL^−1^ streptomycin at OD_730_ = 0.2 in an Erlenmeyer flask equipped with a screw-cap. The transformants with pKT-GSS-KDC-ADH and pKT-GSS-KDC-ADH-CcaR were grown at 30 °C with shaking at 100 rpm under red light illumination alone (non-inductive condition) or under both red and green light illumination (inductive condition). The transformant with pKT-P_*trc-core*_-KDC-ADH was grown at 30 °C with shaking at 100 rpm under both red and green light illumination. The culture supernatant was harvested every day from 2 mL of culture medium by centrifugation at 10,000×*g* for 2 min and then subjected to gas chromatography analysis. The cell pellets were transferred to the same flask. Two milliliters of fresh 500 mM NaHCO_3_ was added to the culture every day after transferring 20 µL of culture into 180 µL of fresh BG11 medium for measurement of OD_730_ using a V-630 UV and visible spectrophotometer (JASCO, Co., Ltd., Tokyo, Japan). The production titers of isobutanol and 3MB in the culture supernatant were detected via gas chromatography analysis, as described below.

The culture supernatants were analyzed using a gas chromatograph GC-2014 (Shimadzu Co., Ltd., Kyoto, Japan) equipped with a flame ionization detector (FID) and a capillary column InertCap^®^ FFAP (60 m, 0.32 mm internal diameter, 1.0-µm film thickness; GL Science Co., Ltd., Tokyo, Japan). The GC oven temperature was maintained at 40 °C for 3 min, increased at a gradient of 30 °C min^−1^ from 40 to 150 °C, further increased at a gradient of 20 °C min^−1^ from 150 to 230 °C, and finally maintained at 230 °C for 1 min. The temperatures of the injector and FID detector were 225 °C and 250 °C, respectively. The concentrations of isobutanol and 3MB in the culture supernatant were determined from a standard curve created using appropriate standards (Tokyo Chemical Industry Co. Ltd., Tokyo, Japan). A 2 mL aliquot of the culture was used to calculate the titer, and 2 mL of fresh NaHCO_3_ solution was added for each sampling. The amount of alcohol in the 2 mL aliquot was taken into account to calculate the titer of the following days.

## Supplementary Information

**Additional file 1: Fig. S1 **Design of vectors used in this study.** (a) **pKT-GSS-KDC-ADH for the application of CcaS/CcaR system to produce alcohol in cyanobacteria. pKT-GSS-KDC-ADH was constructed by inserting *kdc *and *adh *into the downstream of P_*cpcG2*_ on pKT-GSS. **(b) **pKT-GSS-KDC-ADH-CcaR to tune the CcaS/CcaR system up for the enhancement of alcohol production. pKT-GSS-KDC-ADHCcaR was constructed by inserting the structural genes of *kdc*, *adh, *and *ccaR *into the downstream of P_*cpcG2*_ on pKT-GSS. **(c) **pKT-P_*trc-core*_-KDC-ADH for the constitutive activation of alcohol production in cyanobacteria. The *ori*V origin is replicated in *Synechocystis *sp. PCC 6803. Gene abbreviations are as follows: P_*ccaS*_, *ccaS *promoter; P_*ccaR*_, *ccaR *promoter; P_*cpcG2*_, *cpcG2 *promoter; *kdc*, keto-acid decarboxylase; *adh*, alcohol dehydrogenase; Sm^R^, streptomycin-resistance gene; SD_*cpcB*_, Shine Dalgarno-like sequence of *cpcB *derived from *Synechococcus *sp. PCC 7002. **Fig. S2 **Growth curves of each strain used in this study. Growth curves of **(a) **the PCC 6803ΔGSS strain with pKT-GSS-KDC-ADH, **(b) **the PCC 6803ΔGSS strain, and **(c) **the PCC 6803ΔGSS strain with pKT-GSS-KDC-ADH-CcaR. All strains were cultivated under the red light illumination alone (black markers) and under the red and green light illumination (white markers). All analyses were performed in triplicate. Error bars represent the standard deviation (SD) calculated from triplicate experiments using one clone. **Fig. S3 **Transcriptional analysis of *kdc *gene in the *Synechocystis *sp. PCC 6803ΔGSS strain. The relative gene transcription levels of *kdc *in the transformants of *Synechocystis *sp. PCC 6803ΔGSS integrated with pKT-GSS-KDC-ADH cultivated under the red light illumination alone (black bar) and under the red and green light illumination (white bar) are shown. All analyses were performed in triplicate. Error bars represent the SD calculated from triplicate experiments using one clone. **Fig. S4 **Evaluation of alcohol production utilizing the P_*trc-core*_ system in *Synechocystis *sp, PCC 6803ΔGSS.** (a) **Production titer of isobutanol in the culture supernatant of *Synechocystis *sp. PCC 6803ΔGSS transformed with pKT-P_*trc-core*_-KDC-ADH cultivated under the red and green light illumination. **(b) **Specific titer of isobutanol under the red and green light illumination. **(c) **Growth curve of the transformant cultivated under the red and green light illumination. All analyses were performed in triplicate. Error bars represent the SD calculated from triplicate experiments using one clone.** Fig. S5 **Comparison of transcription level of *kdc *gene between the P_*trc-core*_ and the P_*cpcG2*_. Transcription analysis was conducted after 24 h of cultivation of the PCC 6803ΔGSS strain transformed with pKT-P_*trc-core*_-KDC-ADH or pKT-GSS-KDC-ADH under red and green light illumination. All analyses were performed in triplicate. Error bars represent the SD calculated from triplicate experiments using one clone. Statistical analysis was performed using Student’s *t*-test: * p < 0.05. **Fig. S6 **Comparison of transcription level of *kdc *gene from the P_*cpcG2*_ under the various intensities of green light. Transcription level of *kdc *in the PCC 6803ΔGSS strain transformed with pKT-GSSKDC-ADH under green light illumination (0 µmol m^-2^ s^-1^, 1 µmol m^-2^ s^-1^, 5 µmol m^-2^ s^-1^, 30 µmol m^-2^ s^-1^, 50 µmol m^-2^ s^-1^, or 100 µmol m^-2^ s^-1^) with red light illumination (30 µmol m^-2^ s^-1^) was shown. All analyses were performed in triplicate. Error bars represent the SD calculated from triplicate experiments using one clone. Statistical analysis was performed using one-way analysis of variance (ANOVA) with Tukey’s multiple comparison test: * p < 0.05, ** p < 0.005. **Table S1 **Comparison of the isobutanol production titer in cyanobacteria expressing keto-acid decarboxylase (*kdc*) and alcohol dehydrogenase (*adh*) in this study with that in previous studies. **Table S2 **Comparison of the isobutanol production in this study with the highest titer and productivity achieved in previous studies. **Table S3 **Comparison of the 3-methyl-1-butanol titer in cyanobacteria obtained in this study with that in previous studies. **Table S4** Sequences of the vector inserts used in this study. pKT-GSS-KDC-ADH. **Table S5** Primer sequences used in this study.

## Data Availability

All gene sequences used in this study are listed in Additional file 1.

## Abbreviations

ADH: Alcohol dehydrogenase
GSS: Green light sensing system
IPTG: Isopropyl-β-d-thiogalactopyranoside
KDC: Keto-acid decarboxylase
PCC 6803: *Synechocystis* sp. PCC 6803
PCC 7942: *Synechococcus* sp. PCC 7942
3MB: 3-Methyl-1-butanol
