# Supplementary material for: Light-induced production of isobutanol and 3-methyl-1-butanol by metabolically engineered cyanobacteria
Source: Microb Cell Fact. 2022 Jan 6;21:7. doi: 10.1186/s12934-021-01732-x (PMC8740407; doi:10.1186/s12934-021-01732-x)
Supplement: Supplementary file 1 — Additional file 1: Fig. S1 Design of vectors used in this study. (a) pKT-GSS-KDC-ADH for the application of CcaS/CcaR system to produce alcohol in cyanobacteria. pKT-GSS-KDC-ADH was constructed by inserting kdc and adh into the downstream of PcpcG2 on pKT-GSS. (b) pKT-GSS-KDC-ADH-CcaR to tune the CcaS/CcaR system up for the enhancement of alcohol production. pKT-GSS-KDC-ADHCcaR was constructed by inserting the structural genes of kdc, adh, and ccaR into the downstream of PcpcG2 on pKT-GSS. (c) pKT-Ptrc-core-KDC-ADH for the constitutive activation of alcohol production in cyanobacteria. The oriV origin is replicated in Synechocystis sp. PCC 6803. Gene abbreviations are as follows: PccaS, ccaS promoter; PccaR, ccaR promoter; PcpcG2, cpcG2 promoter; kdc, keto-acid decarboxylase; adh, alcohol dehydrogenase; SmR, streptomycin-resistance gene; SDcpcB, Shine Dalgarno-like sequence of cpcB derived from Synechococcus sp. PCC 7002. Fig. S2 Growth curves of each strain used in this study. Growth curves of (a) the PCC 6803ΔGSS strain with pKT-GSS-KDC-ADH, (b) the PCC 6803ΔGSS strain, and (c) the PCC 6803ΔGSS strain with pKT-GSS-KDC-ADH-CcaR. All strains were cultivated under the red light illumination alone (black markers) and under the red and green light illumination (white markers). All analyses were performed in triplicate. Error bars represent the standard deviation (SD) calculated from triplicate experiments using one clone. Fig. S3 Transcriptional analysis of kdc gene in the Synechocystis sp. PCC 6803ΔGSS strain. The relative gene transcription levels of kdc in the transformants of Synechocystis sp. PCC 6803ΔGSS integrated with pKT-GSS-KDC-ADH cultivated under the red light illumination alone (black bar) and under the red and green light illumination (white bar) are shown. All analyses were performed in triplicate. Error bars represent the SD calculated from triplicate experiments using one clone. Fig. S4 Evaluation of alcohol production utilizing the Ptrc-core system in [file 12934_2021_1732_MOESM1_ESM.pdf]

Microbial Cell Factories

Additional file 1

Green light-induced production of isobutanol and 3-methyl-1-butanol by metabolically engineered cyanobacteria

Shunichi Kobayashi[1], Shota Atsumi[2], Kazunori Ikebukuro[1], Koji Sode[3], and Ryutaro Asano[1] §

[1]Department of Biotechnology and Life Science, Tokyo University of Agriculture and Technology, 2-24-16, Naka-cho, Koganei, Tokyo 184-8588, Japan

[2]Department of Chemistry, University of California Davis, One Shields Avenue, Davis, CA 95616, United States

[3]Joint Department of Biomedical Engineering, The University of North Carolina at Chapel Hill and North Carolina State University, Chapel Hill, NC 27599, United States

§ Corresponding author; Ryutaro Asano (ryutaroa@cc.tuat.ac.jp)

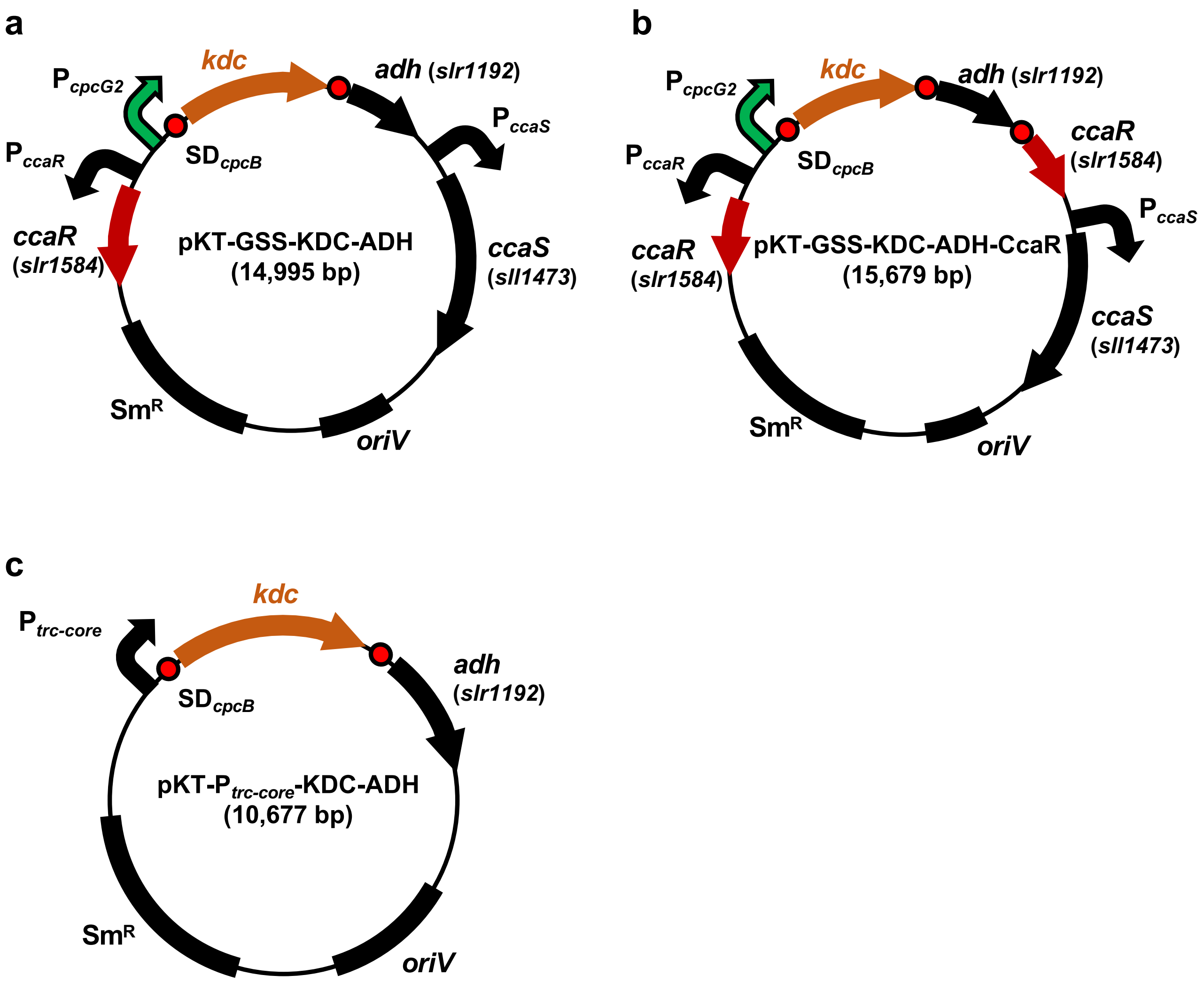

Fig. S1 Design of vectors used in this study. **(a)** pKT-GSS-KDC-ADH for the application of CcaS/CcaR system to produce alcohol in cyanobacteria. pKT-GSS-KDC-ADH was constructed by inserting *kdc* and *adh* into the downstream of P_{cpcG2} on pKT-GSS. **(b)** pKT-GSS-KDC-ADH-CcaR to tune the CcaS/CcaR system up for the enhancement of alcohol production. pKT-GSS-KDC-ADH-CcaR was constructed by inserting the structural genes of *kdc*, *adh,* and *ccaR* into the downstream of P_{cpcG2} on pKT-GSS. **(c)** pKT-$P_{trc\text{-}core}$-KDC-ADH for the constitutive activation of alcohol production in cyanobacteria. The *ori*V origin is replicated in *Synechocystis* sp. PCC 6803. Gene abbreviations are as follows: P_{ccaS}, *ccaS* promoter; P_{ccaR}, *ccaR* promoter; P_{cpcG2}, *cpcG2* promoter; *kdc*, keto-acid decarboxylase; *adh*, alcohol dehydrogenase; Sm^R, streptomycin-resistance gene; SD_{cpcB}, Shine Dalgarno-like sequence of *cpcB* derived from *Synechococcus* sp. PCC 7002.

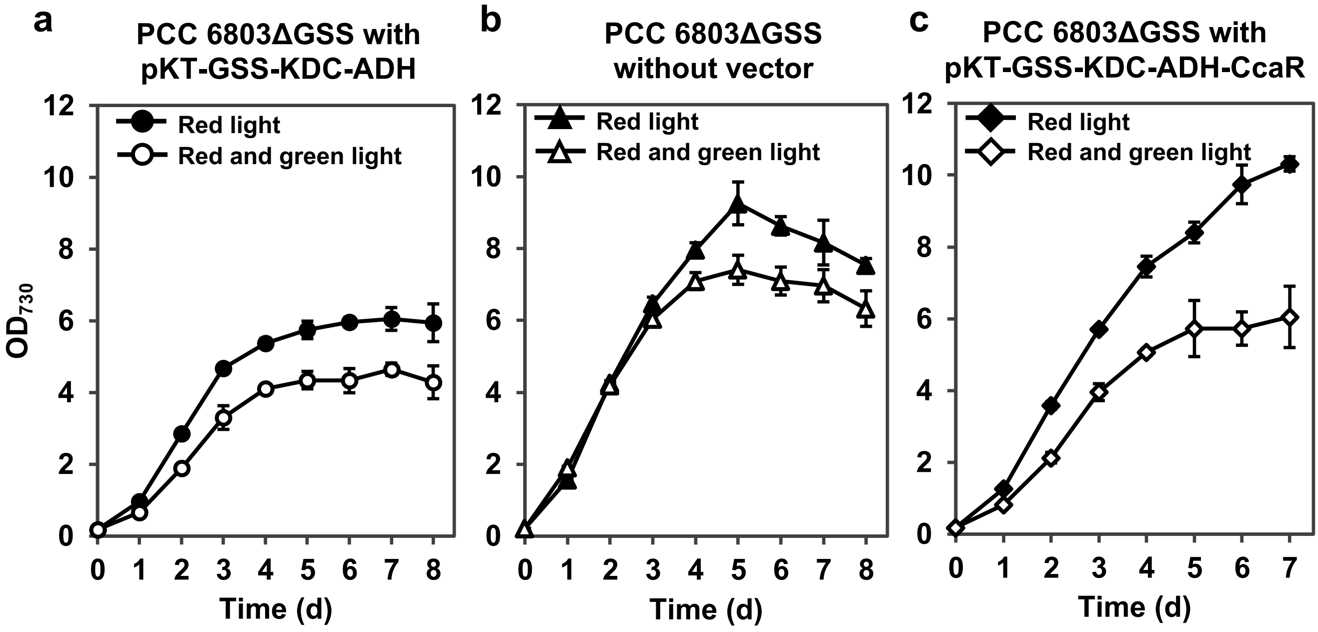

Fig. S2 Growth curves of each strain used in this study. Growth curves of **(a)** the PCC 6803ΔGSS strain with pKT-GSS-KDC-ADH, **(b)** the PCC 6803ΔGSS strain, and **(c)** the PCC 6803ΔGSS strain with pKT-GSS-KDC-ADH-CcaR. All strains were cultivated under the red light illumination alone (black markers) and under the red and green light illumination (white markers). All analyses were performed in triplicate. Error bars represent the standard deviation (SD) calculated from triplicate experiments using one clone.

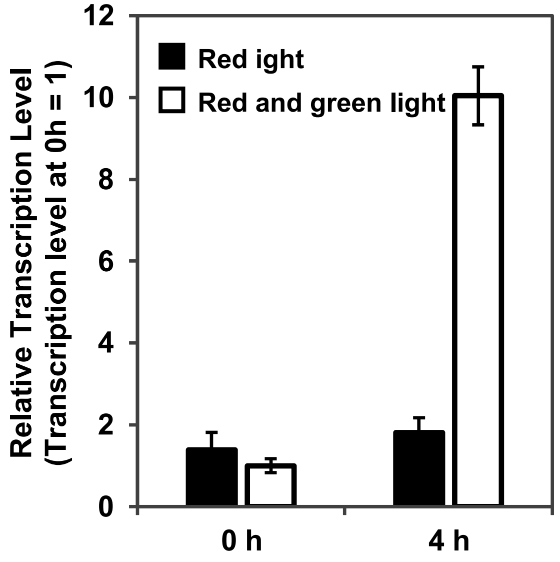

Fig. S3 Transcriptional analysis of *kdc* gene in the *Synechocystis* sp. PCC 6803ΔGSS strain. The relative gene transcription levels of *kdc* in the transformants of *Synechocystis* sp. PCC 6803ΔGSS integrated with pKT-GSS-KDC-ADH cultivated under the red light illumination alone (black bar) and under the red and green light illumination (white bar) are shown. All analyses were performed in triplicate. Error bars represent the SD calculated from triplicate experiments using one clone.

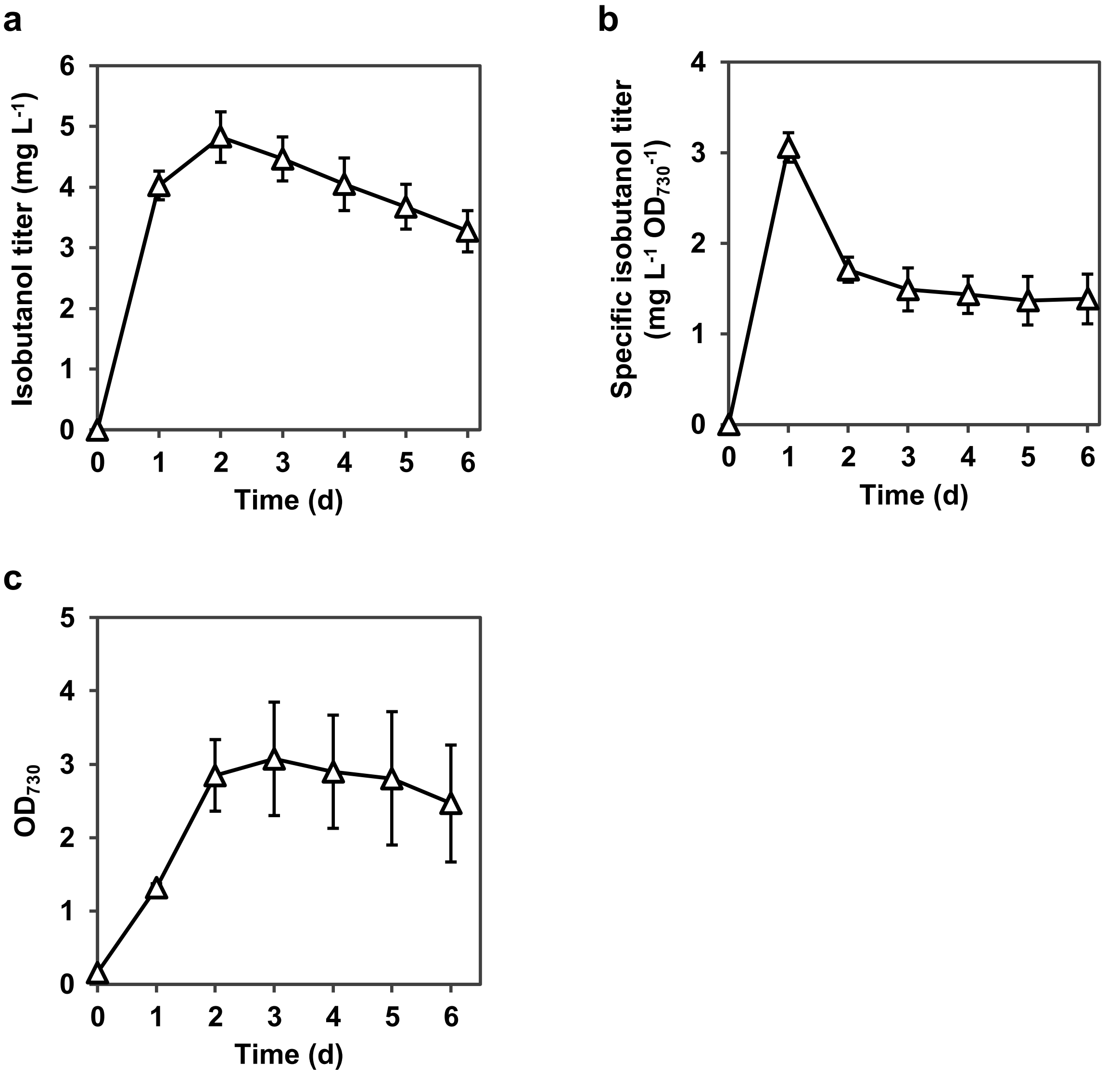

Fig. S4 Evaluation of alcohol production utilizing the $P_{trc\text{-}core}$ system in *Synechocystis* sp, PCC 6803ΔGSS. **(a)** Production titer of isobutanol in the culture supernatant of *Synechocystis* sp. PCC 6803ΔGSS transformed with pKT-$P_{trc\text{-}core}$-KDC-ADH cultivated under the red and green light illumination. **(b)** Specific titer of isobutanol under the red and green light illumination. **(c)** Growth curve of the transformant cultivated under the red and green light illumination. All analyses were performed in triplicate. Error bars represent the SD calculated from triplicate experiments using one clone.

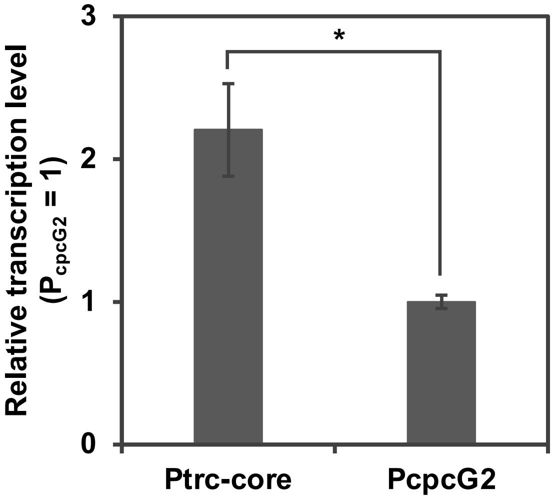

Fig. S5 Comparison of transcription level of *kdc* gene between the $P_{trc\text{-}core}$ and the P_{cpcG2}. Transcription analysis was conducted after 24 h of cultivation of the PCC 6803ΔGSS strain transformed with pKT-$P_{trc\text{-}core}$-KDC-ADH or pKT-GSS-KDC-ADH under red and green light illumination. All analyses were performed in triplicate. Error bars represent the SD calculated from triplicate experiments using one clone. Statistical analysis was performed using Student's *t*-test: * $p < 0.05$.

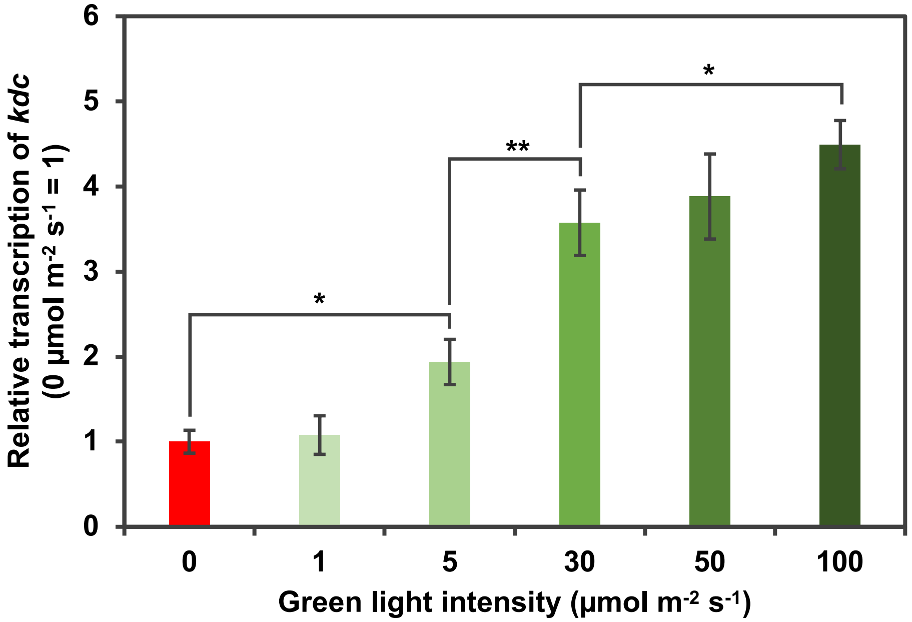

Fig. S6 Comparison of transcription level of *kdc* gene from the P_{cpcG2} under the various intensities of green light. Transcription level of *kdc* in the PCC 6803ΔGSS strain transformed with pKT-GSS-KDC-ADH under green light illumination (0 μmol m^{-2} s^{-1}, 1 μmol m^{-2} s^{-1}, 5 μmol m^{-2} s^{-1}, 30 μmol m^{-2} s^{-1}, 50 μmol m^{-2} s^{-1}, or 100 μmol m^{-2} s^{-1}) with red light illumination (30 μmol m^{-2} s^{-1}) was shown. All analyses were performed in triplicate. Error bars represent the SD calculated from triplicate experiments using one clone. Statistical analysis was performed using one-way analysis of variance (ANOVA) with Tukey's multiple comparison test: * $p < 0.05$, ** $p < 0.005$.

Table S1 Comparison of the isobutanol production titer in cyanobacteria expressing keto-acid decarboxylase (*kdc*) and alcohol dehydrogenase (*adh*) in this study with that in previous studies.

Strain	Promoter (Inducer)	Genes	Titer	Productivity*	Production time	Ref.
PCC 7942	P_{trc} (IPTG)	*kdc*, *yqhD*	50 mg L^{-1}	17 mg L^{-1} d^{-1}	3 d	[3]
PCC 6803	P_{tac} (No inducer)	*kdc*, *adhA*	90 mg L^{-1}	15 mg L^{-1} d^{-1}	6 d	[26]
PCC 6803	$P_{trc\text{-}core}$ (No inducer)	*kdc* (S286T)	911 mg L^{-1}	20 mg L^{-1} d^{-1}	46 d	[28]
PCC 6803ΔGSS	P_{cpcG2} (Green light)	*kdc*, *slr1192*	238 mg L^{-1}	48 mg L^{-1} d^{-1}	5 d	This study

* The productivity was calculated as the average production level per day by dividing the titer by the number of days required for production.

Table S2 Comparison of the isobutanol production in this study with the highest titer and productivity achieved in previous studies.

Strain	Promoter (Inducer)	Genes	Titer	Productivity*	Production time	Ref.
PCC 7942	P_{trc}, P_{LlacO1} (IPTG)	*kdc*, *yqhD*, *alsS*, *ilvC*, *ilvD*	450 mg L^{-1}	75 mg L^{-1} day^{-1}	6 d	[3]
PCC 7942Δ*glgC*	P_{trc}, P_{LlacO1} (IPTG)	*kdc*, *yqhD*, *alsS*, *ilvC*, *ilvD*	550 mg L^{-1}	69 mg L^{-1} day^{-1}	8 d	[24]
PCC 7942	P_{trc}, P_{LlacO1} (IPTG)	*kdc*, *adhA*, *alsS*, *ilvC*, *ilvD*	637 mg L^{-1}	32 mg L^{-1} day^{-1}	20 d	[25]
PCC 6803	$P_{trc\text{-}core}$ (No inducer)	*kdc* (S286T)	911 mg L^{-1}	20 mg L^{-1} day^{-1}	46 d	[28]
PCC 6803ΔGSS	P_{cpcG2} (Green light)	*kdc*, *slr1192*	238 mg L^{-1}	48 mg L^{-1} day^{-1}	5 d	This study

* The productivity was calculated as the average production level per day by dividing the titer by the number of days required for production.

Table S3 Comparison of the 3-methyl-1-butanol titer in cyanobacteria obtained in this study with that in previous studies.

Strain	Promoter (Inducer)	Genes	Titer	Productivity*	Production time	Ref.
PCC 7942Δ*glgC*	P_{trc}, P_{LlacO1} (IPTG)	*kdc*, *yqhD*, *alsS*, *ilvC*, *ilvD*	70 mg L^{-1}	10 mg L^{-1} d^{-1}	7 d	[20]
PCC 6803	$P_{trc\text{-}core}$ (No inducer)	*kdc* (S286T)	225 mg L^{-1}	5 mg L^{-1} d^{-1}	46 d	[23]
PCC 6803ΔGSS	P_{cpcG2} (Green light)	*kdc*, *slr1192*	75 mg L^{-1}	15 mg L^{-1} d^{-1}	5 d	This study

* The productivity was calculated as the average production level per day by dividing the titer by the number of days required for production.

Table S4 Sequences of the vector inserts used in this study.

**pKT-GSS-KDC-ADH**
CTGCAGTGCCACTTGACGTCTAAGAAAAGGAATATTCAGCAATTTGCCCGTGCCGAAGAAAGGCCCACCCGT<br>GAAGGTGAGCCAGTGAGTTGATTGCTACGTAATTAGTTAGTTAGCCCTTAGTGACTCGAATTCGCGGCCGCT<br>TCTAGGCGCAACGTTCAAATCCGCTCCCGGCGGATTTGTCCTACTCAGGAGAGCGTTCACCGACAAACAACA<br>GATAAAACGAAAGGCCCAGTCTTTCGACTGAGCCTTTCGTTTTATTTGATGCCTGGCAGTTCCCTACTCTCG<br>CATGGGGAGACCCCACACTACCATCGGCGCTACGGCGTTTCACTTCTGAGTTCGGCATGGGGTCAGGTGGGA<br>CCACCGCGCTACTGCCGCCAGGCAAATTCTGTTTTATCAGACCGCTTCTGCGTTCTGATTTAATCTGTATCA<br>GGCTGAAAATCTTCTCTCATCCGCCAAAACAGCCAAGCTTCTAGTTTTTCCCTTGGCACAAAGATTTTTCCG<br>TTAAATTGGCCAGACGATACCCAATGCCATGGACCGTTTCAATGGCATCTGCTGATAAACCGGCACTTTTTA<br>ATTTTTGTCGCAAACTCCGCACATGCACCTTAACCGTATCTTCCTCTGGGGGACTCTCCAACTTCCAGATAC<br>TATCGATAATCATGCTCCGACTTAGCACCCGACGGCCATTGCGGAGTAGTAATTCCAGAATGCTGTATTCCT<br>TGCGGGTCAAAGACAAAACCTCATTGTCATAACTAACTTCATAGGTGCTTGGATCCAACCTGATTGGCCCCC<br>ACTCTAAAACTGGTTGGCACGTTGCACAACCCCGACGCAACAAAGCTCGCACCCTGGCAAATAACTCCCCCA<br>AATCCACTGGCTTGACCACATAATCATCCGCCCCCGCATCCAAGCCCGTGATTTTATCATTGATCGTATCCC<br>TGGCTGTCATCATCAAAATTGGCATTAAATAACTGTGCGATCGCCATTTTTGACAGAGGGTAATCCCGTCCA<br>ACTCCGGCAGCATCACATCCAAAATAACGAGGTCATATTCCAGTCGGGAGGCATAGTCCCAGGCGAGGGAAG<br>CGTCGGTGGCAATATCAACGGTGTAAAGCTGGTCACTCAATGCTTCAGCAAGGGTTTCCGCCAGCGGCAAAT<br>CATCCTCCACTAAAAGAATTCTCATAGCCCATTGTGCTTTTCTCTATCAACCTCAGCTTACCTGAAGGGGTG<br>AACAGGTCTGGGTTAATTCATGTTGCGAAATGTAACAGTTTTAGTCGCATCAGCTAACTTTCCGATTTCTTT<br>ACGATTTTCTCCCCCTTTTCTTCAATTTTACTTTGTTAGGATCGCATTTTTAATGCCAACACATACCAGTTA<br>TTGGCTGGACATTAAACAACTTTTAAGTTTAATTACTAACTAGATCTTATAAGTAGGAGATAAAAACATGTA<br>TACGGTTGGAGATTATCTGCTTGATCGCCTGCATGAACTAGGCATTGAGGAAATCTTTGGTGTTCCGGGTGA<br>TTACAATCTGCAATTTCTGGATCAGATCATTAGTCGCAAAGACATGAAATGGGTTGGTAACGCCAATGAACT<br>GAATGCGTCGTATATGGCTGACGGTTATGCCCGTACCAAAAAAGCGGCTGCGTTCTTGACGACCTTTGGTGT<br>GGGAGAACTTAGCGCCGTAAATGGCTTAGCCGGCAGCTATGCGGAAAACCTGCCTGTAGTGGAAATCGTTGG<br>CTCTCCCACGTCAAAAGTGCAGAATGAAGGCAAATTCGTCCATCATACCCTGGCAGATGGCGATTTTAAGCA<br>CTTTATGAAAATGCATGAACCTGTGACAGCGGCGAGAACTCTGTTAACCGCGGAAAACGCCACCGTTGAGAT<br>CGACCGCGTACTAAGCGCTCTGCTTAAAGAACGTAAACCGGTGTATATCAATCTGCCGGTAGATGTCGCGGC<br>TGCGAAAGCCGAAAAACCAAGTTTACCGCTGAAGAAAGAGAATAGCACATCTAACACCAGTGATCAGGAAAT<br>CCTCAACAAGATCCAGGAATCCCTGAAAAATGCGAAAAAGCCGATCGTGATTACAGGACACGAAATTATTTC<br>ATTTGGGCTGGAGAAAACCGTGTCTCAGTTCATAAGCAAGACGAAACTGCCTATTACCACCTTGAACTTTGG<br>TAAATCGTCCGTGGATGAAGCGTTACCGTCCTTTTTGGGGATTTACAATGGCAAACTGTCAGAGCCGAATTT<br>GAAGGAATTCGTGGAAAGCGCCGATTTCATTCTGATGCTTGGCGTGAAACTGACTGATAGTTCGACGGGTGC<br>CTTTACTCATCACTTAAACGAGAACAAAATGATCTCTCTCAACATTGACGAGGGCAAGATTTTCAACGAGTC<br>CATTCAGAACTTCGACTTCGAGTCTTTGATTAGCAGTCTGCTGGATCTGTCGGAAATCGAATACAAAGGGAA<br>ATACATTGACAAGAAACAGGAAGACTTTGTACCGTCCAATGCATTACTGAGTCAAGATCGCCTGTGGCAAGC<br>GGTTGAGAACCTTACGCAAAGCAACGAAACCATAGTCGCCGAACAAGGTACGTCGTTTTTCGGAGCAAGCAG<br>CATCTTCCTGAAACCCAAATCACACTTTATCGGTCAACCACTCTGGGGTTCTATTGGTTACACCTTTCCAGC<br>AGCTCTCGGCTCACAGATAGCGGATAAAGAGAGTCGACACCTGCTGTTTATTGGCGATGGTTCTTTGCAGTT<br>GACTGTTCAGGAACTCGGCTTAGCAATTCGGGAGAAGATTAACCCCATTTGCTTCATCATCAATAATGACGG<br>GTATACCGTCGAACGTGAAATCCATGGGCCAAATCAAAGCTATAACGACATTCCGATGTGGAACTATTCGAA<br>ACTTCCGGAATCGTTTGGCGCAACTGAAGAACGTGTCGTGTCCAAAATCGTGCGTACAGAAAACGAGTTTGT<br>CAGCGTTATGAAAGAAGCGCAGGCAGATCCGAATCGCATGTACTGGATCGAGCTGATTCTGGCCAAAGAAGA<br>CGCTCCGAAAGTCCTGAAGAAAATGGGCAAACTCTTCGCAGAACAGAACAAATCCTAATATAAGTAGGAGAT<br>AAAAACATGATTAAAGCCTACGCTGCCCTGGAAGCCAACGGAAAACTCCAACCCTTTGAATACGACCCCGGT<br>GCCCTGGGTGCTAATGAGGTGGAGATTGAGGTGCAGTATTGTGGGGTGTGCCACAGTGATTTGTCCATGATT<br>AATAACGAATGGGGCATTTCCAATTACCCCCTAGTGCCGGGTCATGAGGTGGTGGGTACTGTGGCCGCCATG<br>GGCGAAGGGGTGAACCATGTTGAGGTGGGGGATTTAGTGGGGCTGGGTTGGCATTCGGGCTACTGCATGACC<br>TGCCATAGTTGTTTATCTGGCTACCACAACCTTTGTGCCACGGCGGAATCGACCATTGTGGGCCACTACGGT<br>GGCTTTGGCGATCGGGTTCGGGCCAAGGGAGTCAGCGTGGTGAAATTACCTAAAGGCATTGACCTAGCCAGT<br>GCCGGGCCCCTTTTCTGTGGAGGAATTACCGTTTTCAGTCCTATGGTGGAACTGAGTTTAAAGCCCACTGCA<br>AAAGTGGCAGTGATCGGCATTGGGGGCTTGGGCCATTTAGCGGTGCAATTTCTCCGGGCCTGGGGCTGTGAA<br>GTGACTGCCTTTACCTCCAGTGCCAGGAAGCAAACGGAAGTGTTGGAATTGGGCGCTCACCACATACTAGAT<br>TCCACCAATCCAGAGGCGATCGCCAGTGCGGAAGGCAAATTTGACTATATTATCTCCACTGTGAACCTGAAG<br>CTTGACTGGAACTTATACATCAGCACCCTGGCGCCCCAGGGACATTTCCACTTTGTTGGGGTGGTGTTGGAG

CCTTTGGATCTAAATCTTTTTCCCCTTTTGATGGGACAACGCTCCGTTTCTGCCTCCCCAGTGGGTAGTCCC
GCCACCATTGCCACCATGTTGGACTTTGCTGTGCGCCATGACATTAAACCCGTGGTGGAACAATTTAGCTTT
GATCAGATCAACGAGGCGATCGCCCATCTAGAAAGCGGCAAAGCCCATTATCGGGTAGTGCTCAGCCATAGT
AAAAATTAGTCTAGATTTTTTTGACGGTAAAGCCAAGCTGAAGGCTGACAAAATCTATACTGTTATATCCCC
CTTTCCTGGGGGACTTAGGCGGTTTAATAACTGGGGTTTTTGCTAGAAATATTTTGGCGTTTAATTATTTAT
TCTCTGGTTTAATATTTCGAGACTTTGCAGACTTTGTATTTGAGGCCAGACTCCAATGTTGAAACTAGCCGG
TAAAAAAGCCTCTGCCCTGACCTGCAAGAAAACGAAGGCAAAAATTGGTATCCTAGAAATGGATCGGCAGTG
GTATCAATCAGTCAAGCGATTTTTATGTCCAAGTTTTGAAATTTCTGCTATCAACTTCCAAGATATTTTGTT
TAACAATGTTGACGTTGGAGAATATCATTCGATCCTAGTTGGTTGCGGGATTAAATATGAATCAATTGATAT
TTCAGCTACTTTAGATTTAGTGACCATTATTAAAAAATTGTCTGCTAAGCCGCCGGCCCTACTACTAATTAC
GGATTGTGCCGACAGTCAGATCACGGTACAAGCTCGCAGTTACCTGCCCCAAATTGACGGCGTTTTTGCCAA
GGATCATGATTTAGCTTTATTGTTAAAAGTCATGAAAATTATTGCCAAACAAAAATATTTTAAGTAAGGTTT
TTCTACTGGGATTCGCTAATATATGGGCAAATTTCTAATTCCAATCGAATTTGTTTTTCTGGCGATCGCCAT
GACCTGTTATTTATGGCACAGACAAAACCAAGAACGCCGCAGGATTGAAATTAGCATCAAGCAACAAACCCA
ACGGGAACGATTTATTAACCAAATTACCCAACATATCCGCCAATCTTTAAACTTGGAAACGGTTTTAAATAC
CACCGTCGCTGAAGTTAAAACCCTGTTGCAAGTTGATCGAGTTCTAATTTATCGCATTTGGCAAGATGGCAC
GGGCAGCGCCATTACGGAATCGGTGAATGCCAATTATCCTAGTATTTTAGGGCGGACCTTTTCCGATGAAGT
TTTTCCCGTTGAATACCATCAAGCCTACACCAAAGGTAAAGTACGGGCCATTAATGACATTGACCAGGATGA
CATAGAGATTTGCCTAGCTGATTTCGTCAAACAATTTGGCGTGAAATCAAAATTAGTAGTGCCCATTCTTCA
ACATAATCGTGCTTCTTCCCTAGATAATGAATCAGAATTTCCCTATCTTTGGGGGCTGTTAATTACCCATCA
ATGTGCTTTTACCCGGCCATGGCAACCGTGGGAAGTGGAGTTAATGAAACAGCTAGCCAATCAGGTCGCGAT
CGCCATCCAACAATCGGAATTATATGAGCAATTACAGCAACTCAATAAAGATTTGGAAAACCGAGTCGAAAA
ACGCACCCAGCAACTTGCCGCCACCAATCAATCCCTAAGAATGGAAATCAGTGAGCGACAAAAAACGGAAGC
CGCTCTCCGCCACACTAACCATACTCTGCAATCCCTGATTGCGGCCTCCCCCAGGGGTATTTTTACCCTTAA
TTTAGCAGACCAAATTCAGATTTGGAATCCTACAGCAGAACGTATTTTTGGTTGGACAGAAACAGAAATTAT
TGCCCATCCAGAATTATTAACATCCAACATTTTGCTGGAAGATTATCAGCAATTTAAACAGAAAGTTTTATC
AGGCATGGTTTCCCCTAGCCTAGAATTAAAATGTCAAAAAAAAGATGGTAGTTGGATTGAAATTGTCCTTTC
CGCTGCTCCCCTATTGGATAGTGAAGAAAATATTGCCGGATTGGTGGCGGTTGTCGCCGATATTACCGAGCA
AAAGCGGCAGGCAGAACAAATTCGTTTGCTACAATCCGTTGTGGTTAATACTAATGATGCGGTGGTGATTAC
GGAAGCGGAGCCCATTGATGATCCCGGGCCGAGAATTCTCTATGTCAATGAAGCATTTACTAAAATCACCGG
TTATACTGCTGAAGAAATGCTAGGCAAAACCCCCCGAGTTTTACAGGGACCAAAAACTAGTCGCACTGAATT
AGATAGGGTGCGGCAAGCCATTAGTCAATGGCAATCAGTTACCGTTGAAGTGATTAATTATCGTAAGGATGG
CAGTGAGTTTTGGGTGGAATTTAGTCTGGTGCCCGTTGCCAATAAAACAGGTTTTTACACCCATTGGATTGC
TGTGCAAAGGGATGTCACTGAGCGCCGACGCACGGAGGAAGTCCGCCTAGCTTTAGAACGGAAAAAGAATT
AAGCCGCCTAAAAACTCGTTTTTTCTCCATGGCTTCCCATGAATTTCGTACTCCCCTCAGTACGGCCTTAGC
TGCTGCCCAATTACTGGAAAATTCTGAAGTGGCCTGGCTTGATCCCGATAAGCGTAGCCGGAACTTACACCG
TATTCAAAATTCCGTGAAAAATATGGTACAGCTCCTGGATGATATTTTAATCATTAACCGTGCCGAAGCGGG
CAAATTGGAATTTAATCCTAATTGGTTAGATTTGAAATTATTGTTCCAGCAATTTATCGAAGAAATTCAATT
AAGTGTCAGTGACCAATATTATTTTGACTTTATTTGTAGCGCTCAAGATACGAAGGCATTGGTGGATGAAAG
GTTAGTGCGGTCTATTTTATCTAATCTGTTATCTAATGCGATTAAATACTCTCCCGGGGGAGGGCAGATTAA
AATTGCCCTAAGCCTAGATTCGGAACAGATTATTTTTGAAGTCACCGACCAGGGCATTGGCATTTCGCCAGA
GGACCAAAAGCAAATTTTTGAACCCTTTCATCGGGGCAAAAATGTCAGAAATATTACGGGAACAGGACTCGG
TTTAATGGTTGCCAAGAAATGTGTTGACTTACACAGTGGCAGTATCTTGCTAAAAAGTGCAGTTGACCAGGG
AACAACAGTTACTATCTGTTTAAAACGCTATAACCATTTGCCTCGAGCTTAGCGCAAAAAACCCCGCTTCGG
CGGGGTTTTTTCGCCTGCAG

pKT-GSS-KDC-ADH-CcaR

CTGCAGTGCCACTTGACGTCTAAGAAAAGGAATATTCAGCAATTTGCCCGTGCCGAAGAAAGGCCCACCCGT
GAAGGTGAGCCAGTGAGTTGATTGCTACGTAATTAGTTAGTTAGCCCTTAGTGACTCGAATTCGCGGCCGCT
TCTAGGCGCAACGTTCAAATCCGCTCCCGGCGGATTTGTCCTACTCAGGAGAGCGTTCACCGACAAACAACA
GATAAAACGAAAGGCCCAGTCTTTCGACTGAGCCTTTCGTTTTATTTGATGCCTGGCAGTTCCCTACTCTCG
CATGGGGAGACCCCACACTACCATCGGCGCTACGGCGTTTCACTTCTGAGTTCGGCATGGGGTCAGGTGGGA
CCACCGCGCTACTGCCGCCAGGCAAATTCTGTTTTATCAGACCGCTTCTGCGTTCTGATTTAATCTGTATCA
GGCTGAAAATCTTCTCTCATCCGCCAAAACAGCCAAGCTTCTAGTTTTTCCCTTGGCACAAAGATTTTTCCG
TTAAATTGGCCAGACGATACCCAATGCCATGGACCGTTTCAATGGCATCTGCTGATAAACCGGCACTTTTTA
ATTTTTGTCGCAAACTCCGCACATGCACCTTAACCGTATCTTCCTCTGGGGGACTCTCCAACTTCCAGATAC
TATCGATAATCATGCTCCGACTTAGCACCCGACGGCCATTGCGGAGTAGTAATTCCAGAATGCTGTATTCCT
TGCGGGTCAAAGACAAAACCTCATTGTCATAACTAACTTCATAGGTGCTTGGATCCAACCTGATTGGCCCCC

ACTCTAAAACTGGTTGGCACGTTGCACAACCCCGACGCAACAAAGCTCGCACCCTGGCAAATAACTCCCCCA
AATCCACTGGCTTGACCACATAATCATCCGCCCCCGCATCCAAGCCCGTGATTTTATCATTGATCGTATCCC
TGGCTGTCATCATCAAAATTGGCATTAAATAACTGTGCGATCGCCATTTTTGACAGAGGGTAATCCCGTCCA
ACTCCGGCAGCATCACATCCAAAATAACGAGGTCATATTCCAGTCGGGAGGCATAGTCCCAGGCGAGGGAAG
CGTCGGTGGCAATATCAACGGTGTAAAGCTGGTCACTCAATGCTTCAGCAAGGGTTTCCGCCAGCGGCAAAT
CATCCTCCACTAAAAGAATTCTCATAGCCCATTGTGCTTTTCTCTATCAACCTCAGCTTACCTGAAGGGGTG
AACAGGTCTGGGTTAATTCATGTTGCGAAATGTAACAGTTTTAGTCGCATCAGCTAACTTTCCGATTTCTTT
ACGATTTTCTCCCCCTTTTCTTCAATTTTACTTTGTTAGGATCGCATTTTTAATGCCAACACATACCAGTTA
TTGGCTGGACATTAAACAACTTTTAAGTTTAATTACTAACTAGATCTTATAAGTAGGAGATAAAAACATGTA
TACGGTTGGAGATTATCTGCTTGATCGCCTGCATGAACTAGGCATTGAGGAAATCTTTGGTGTTCCGGGTGA
TTACAATCTGCAATTTCTGGATCAGATCATTAGTCGCAAAGACATGAAATGGGTTGGTAACGCCAATGAACT
GAATGCGTCGTATATGGCTGACGGTTATGCCCGTACCAAAAAAGCGGCTGCGTTCTTGACGACCTTTGGTGT
GGGAGAACTTAGCGCCGTAAATGGCTTAGCCGGCAGCTATGCGGAAAACCTGCCTGTAGTGGAAATCGTTGG
CTCTCCCACGTCAAAAGTGCAGAATGAAGGCAAATTCGTCCATCATACCCTGGCAGATGGCGATTTTAAGCA
CTTTATGAAAATGCATGAACCTGTGACAGCGGCGAGAACTCTGTTAACCGCGGAAAACGCCACCGTTGAGAT
CGACCGCGTACTAAGCGCTCTGCTTAAAGAACGTAAACCGGTGTATATCAATCTGCCGGTAGATGTCGCGGC
TGCGAAAGCCGAAAAACCAAGTTTACCGCTGAAGAAAGAGAATAGCACATCTAACACCAGTGATCAGGAAAT
CCTCAACAAGATCCAGGAATCCCTGAAAAATGCGAAAAAGCCGATCGTGATTACAGGACACGAAATTATTTC
ATTTGGGCTGGAGAAAACCGTGTCTCAGTTCATAAGCAAGACGAAACTGCCTATTACCACCTTGAACTTTGG
TAAATCGTCCGTGGATGAAGCGTTACCGTCCTTTTTGGGGATTTACAATGGCAAACTGTCAGAGCCGAATTT
GAAGGAATTCGTGGAAAGCGCCGATTTCATTCTGATGCTTGGCGTGAAACTGACTGATAGTTCGACGGGTGC
CTTTACTCATCACTTAAACGAGAACAAAATGATCTCTCTCAACATTGACGAGGGCAAGATTTTCAACGAGTC
CATTCAGAACTTCGACTTCGAGTCTTTGATTAGCAGTCTGCTGGATCTGTCGGAAATCGAATACAAAGGGAA
ATACATTGACAAGAAACAGGAAGACTTTGTACCGTCCAATGCATTACTGAGTCAAGATCGCCTGTGGCAAGC
GGTTGAGAACCTTACGCAAAGCAACGAAACCATAGTCGCCGAACAAGGTACGTCGTTTTTCGGAGCAAGCAG
CATCTTCCTGAAACCCAAATCACACTTTATCGGTCAACCACTCTGGGGTTCTATTGGTTACACCTTTCCAGC
AGCTCTCGGCTCACAGATAGCGGATAAAGAGAGTCGACACCTGCTGTTTATTGGCGATGGTTCTTTGCAGTT
GACTGTTCAGGAACTCGGCTTAGCAATTCGGGAGAAGATTAACCCCATTTGCTTCATCATCAATAATGACGG
GTATACCGTCGAACGTGAAATCCATGGCCAAATCAAAGCTATAACGACATTCCGATGTGGAACTATTCGAA
ACTTCCGGAATCGTTTGGCGCAACTGAAGAACGTGTCGTGTCCAAAATCGTGCGTACAGAAAACGAGTTTGT
CAGCGTTATGAAAGAAGCGCAGGCAGATCCGAATCGCATGTACTGGATCGAGCTGATTCTGGCCAAAGAAGA
CGCTCCGAAAGTCCTGAAGAAAATGGGCAAACTCTTCGCAGAACAGAACAAATCCTAATATAAGTAGGAGAT
AAAAACATGATTAAAGCCTACGCTGCCCTGGAAGCCAACGGAAAACTCCAACCCTTTGAATACGACCCCGGT
GCCCTGGGTGCTAATGAGGTGGAGATTGAGGTGCAGTATTGTGGGGTGTGCCACAGTGATTTGTCCATGATT
AATAACGAATGGGGCATTTCCAATTACCCCCTAGTGCCGGGTCATGAGGTGGTGGGTACTGTGGCCGCCATG
GGCGAAGGGGTGAACCATGTTGAGGTGGGGGATTTAGTGGGGCTGGGTTGGCATTCGGGCTACTGCATGACC
TGCCATAGTTGTTTATCTGGCTACCACAACCTTTGTGCCACGGCGGAATCGACCATTGTGGGCCACTACGGT
GGCTTTGGCGATCGGGTTCGGGCCAAGGGAGTCAGCGTGGTGAAATTACCTAAAGGCATTGACCTAGCCAGT
GCCGGGCCCCTTTTCTGTGGAGGAATTACCGTTTTCAGTCCTATGGTGGAACTGAGTTTAAAGCCCACTGCA
AAAGTGGCAGTGATCGGCATTGGGGGCTTGGGCCATTTAGCGGTGCAATTTCTCCGGGCCTGGGGCTGTGAA
GTGACTGCCTTTACCTCCAGTGCCAGGAAGCAAACGGAAGTGTTGGAATTGGGCGCTCACCACATACTAGAT
TCCACCAATCCAGAGGCGATCGCCAGTGCGGAAGGCAAATTTGACTATATTATCTCCACTGTGAACCTGAAG
CTTGACTGGAACTTATACATCAGCACCCTGGCGCCCCAGGGACATTTCCACTTTGTTGGGGTGGTGTTGGAG
CCTTTGGATCTAAATCTTTTTCCCCTTTTGATGGGACAACGCTCCGTTTCTGCCTCCCCAGTGGGTAGTCCC
GCCACCATTGCCACCATGTTGGACTTTGCTGTGCGCCATGACATTAAACCCGTGGTGGAACAATTTAGCTTT
GATCAGATCAACGAGGCGATCGCCCATCTAGAAAGCGGCAAAGCCCATTATCGGGTAGTGCTCAGCCATAGT
AAAAATTAGTATAAGTAGGAGATAAAAACATGAGAATTCTTTTAGTGGAGGATGATTTGCCGCTGGCGGAAA
CCCTTGCTGAAGCATTGAGTGACCAGCTTTACACCGTTGATATTGCCACCGACGCTTCCCTCGCCTGGGACT
ATGCCTCCCGACTGGAATATGACCTCGTTATTTTGGATGTGATGCTGCCGGAGTTGGACGGATTACCCTCT
GTCAAAAATGGCGATCGCACAGTTATTTAATGCCAATTTTGATGATGACAGCCAGGGATACGATCAATGATA
AAATCACGGGCTTGGATGCGGGGGCGGATGATTATGTGGTCAAGCCAGTGGATTTGGGGGAGTTATTTGCCA
GGGTGCGAGCTTTGTTGCGTCGGGGTTGTGCAACGTGCCAACCAGTTTTAGAGTGGGGGCCAATCAGGTTGG
ATCCAAGCACCTATGAAGTTAGTTATGACAATGAGGTTTTGTCTTTGACCCGCAAGGAATACAGCATTCTGG
AATTACTACTCCGCAATGGCCGTCGGGTGCTAAGTCGGAGCATGATTATCGATAGTATCTGGAAGTTGGAGA
GTCCCCCAGAGGAAGATACGGTTAAGGTGCATGTGCGGAGTTTGCGACAAAAATTAAAAGTGCCGGTTTAT
CAGCAGATGCCATTGAAACGGTCCATGGCATTGGGTATCGTCTGGCCAATTTAACGGAAAAATCTTTGTGCC
AAGGGAAAAACTAGTCTAGATTTTTTTGACGGTAAAGCCAAGCTGAAGGCTGACAAAATCTATACTGTTATA

TCCCCCTTTCCTGGGGGACTTAGGCGGTTTAATAACTGGGGTTTTTGCTAGAAATATTTTGGCGTTTAATTA
TTTATTCTCTGGTTTAATATTTCGAGACTTTGCAGACTTTGTATTTGAGGCCAGACTCCAATGTTGAAACTA
GCCGGTAAAAAAGCCTCTGCCCTGACCTGCAAGAAAACGAAGGCAAAAATTGGTATCCTAGAAATGGATCGG
CAGTGGTATCAATCAGTCAAGCGATTTTTATGTCCAAGTTTTGAAATTTCTGCTATCAACTTCCAAGATATT
TTGTTTAACAATGTTGACGTTGGAGAATATCATTCGATCCTAGTTGGTTGCGGGATTAAATATGAATCAATT
GATATTTCAGCTACTTTAGATTTAGTGACCATTATTAAAAAATTGTCTGCTAAGCCGCCGGCCCTACTACTA
ATTACGGATTGTGCCGACAGTCAGATCACGGTACAAGCTCGCAGTTACCTGCCCCAAATTGACGGCGTTTTT
GCCAAGGATCATGATTTAGCTTTATTGTTAAAAGTCATGAAAATTATTGCCAAACAAAAATATTTTAAGTAA
GGTTTTTCTACTGGGATTCGCTAATATATGGGCAAATTTCTAATTCCAATCGAATTTGTTTTTCTGGCGATC
GCCATGACCTGTTATTTATGGCACAGACAAAACCAAGAACGCCGCAGGATTGAAATTAGCATCAAGCAACAA
ACCCAACGGGAACGATTTATTAACCAAATTACCCAACATATCCGCCAATCTTTAAACTTGGAAACGGTTTTA
AATACCACCGTCGCTGAAGTTAAAACCCTGTTGCAAGTTGATCGAGTTCTAATTTATCGCATTTGGCAAGAT
GGCACGGGCAGCGCCATTACGGAATCGGTGAATGCCAATTATCCTAGTATTTTAGGGCGGACCTTTTCCGAT
GAAGTTTTTCCCGTTGAATACCATCAAGCCTACACCAAAGGTAAAGTACGGGCCATTAATGACATTGACCAG
GATGACATAGAGTTTGCCTAGCTGATTTCGTCAAACAATTTGGCGTGAAATCAAAATTAGTAGTGCCCATTC
TTCAACATAATCGTGCTTCTTCCCTAGATAATGAATCAGAATTTCCCTATCTTTGGGGGCTGTTAATTACCC
ATCAATGTGCTTTTACCCGGCCATGGCAACCGTGGGAAGTGGAGTTAATGAAACAGCTAGCCAATCAGGTCG
CGATCGCCATCCAACAATCGGAATTATATGAGCAATTACAGCAACTCAATAAAGATTTGGAAAACCGAGTCG
AAAAACGCACCCAGCAACTTGCCGCCACCAATCAATCCCTAAGAATGGAAATCAGTGAGCGACAAAAAACGG
AAGCCGCTCTCCGCCACACTAACCATACTCTGCAATCCCTGATTGCGGCCTCCCCCAGGGGTATTTTTACCC
TTAATTTAGCAGACCAAATTCAGATTTGGAATCCTACAGCAGAACGTATTTTTGGTTGGACAGAAACAGAAA
TTATTGCCCATCCAGAATTATTAACATCCAACATTTTGCTGGAAGATTATCAGCAATTTAAACAGAAAGTTT
TATCAGGCATGGTTTCCCCTAGCCTAGAATTAAAATGTCAAAAAAAAGATGGTAGTTGGATTGAAATTGTCC
TTTCCGCTGCTCCCCTATTGGATAGTGAAGAAAATATTGCCGGATTGGTGGCGGTTGTCGCCGATATTACCG
AGCAAAAGCGGCAGGCAGAACAAATTCGTTTGCTACAATCCGTTGTGGTTAATACTAATGATGCGGTGGTGA
TTACGGAAGCGGAGCCCATTGATGATCCCGGGCCGAGAATTCTCTATGTCAATGAAGCATTTACTAAAATCA
CCGGTTATACTGCTGAAGAAATGCTAGGCAAAACCCCCCGAGTTTTACAGGGACCAAAAACTAGTCGCACTG
AATTAGATAGGGTGCGGCAAGCCATTAGTCAATGGCAATCAGTTACCGTTGAAGTGATTAATTATCGTAAGG
ATGGCAGTGAGTTTTGGGTGGAATTTAGTCTGGTGCCCGTTGCCAATAAAACAGGTTTTTACACCCATTGGA
TTGCTGTGCAAAGGGATGTCACTGAGCGCCGACGCACGGAGGAAGTCCGCCTAGCTTTAGAACGGGAAAAAG
AATTAAGCCGCCTAAAAACTCGTTTTTTCTCCATGGCTTCCCATGAATTTCGTACTCCCCTCAGTACGGCCT
TAGCTGCTGCCCAATTACTGGAAAATTCTGAAGTGGCCTGGCTTGATCCCGATAAGCGTAGCCGGAACTTAC
ACCGTATTCAAAATTCCGTGAAAAATATGGTACAGCTCCTGGATGATATTTTAATCATTAACCGTGCCGAAG
CGGGCAAATTGGAATTTAATCCTAATTGGTTAGATTTGAAATTATTGTTCCAGCAATTTATCGAAGAAATTC
AATTAAGTGTCAGTGACCAATATTATTTTGACTTTATTTGTAGCGCTCAAGATACGAAGGCATTGGTGGATG
AAAGGTTAGTGCGGTCTATTTTATCTAATCTGTTATCTAATGCGATTAAATACTCTCCCGGGGGAGGGCAGA
TTAAAATTGCCCTAAGCCTAGATTCGGAACAGATTATTTTTGAAGTCACCGACCAGGGCATTGGCATTTCGC
CAGAGGACCAAAAGCAAATTTTTGAACCCTTTCATCGGGGCAAAAATGTCAGAAATATTACGGGAACAGGAC
TCGGTTTAATGGTTGCCAAGAAATGTGTTGACTTACACAGTGGCAGTATCTTGCTAAAAAGTGCAGTTGACC
AGGGAACAACAGTTACTATCTGTTTAAAACGCTATAACCATTTGCCTCGAGCTTAGCGCAAAAAACCCCGCT
TCGGCGGGGTTTTTTCGCCTGCAG

pKT-$P_{trc-core}$-KDC-ADH

CTGCAGGAGCTGTTGACAATTAATCATCCGGCTCGTATAATGTGTGGAAGATCTTATAAGTAGGAGATAAAA
ACATGTATACGGTTGGAGATTATCTGCTTGATCGCCTGCATGAACTAGGCATTGAGGAAATCTTTGGTGTTC
CGGGTGATTACAATCTGCAATTTCTGGATCAGATCATTAGTCGCAAAGACATGAAATGGGTTGGTAACGCCA
ATGAACTGAATGCGTCGTATATGGCTGACGGTTATGCCCGTACCAAAAAAGCGGCTGCGTTCTTGACGACCT
TTGGTGTGGGAGAACTTAGCGCCGTAAATGGCTTAGCCGGCAGCTATGCGGAAAACCTGCCTGTAGTGGAAA
TCGTTGGCTCTCCCACGTCAAAAGTGCAGAATGAAGGCAAATTCGTCCATCATACCCTGGCAGATGGCGATT
TTAAGCACTTTATGAAAATGCATGAACCTGTGACAGCGGCGAGAACTCTGTTAACCGCGGAAAACGCCACCG
TTGAGATCGACCGCGTACTAAGCGCTCTGCTTAAAGAACGTAAACCGGTGTATATCAATCTGCCGGTAGATG
TCGCGGCTGCGAAAGCCGAAAAACCAAGTTTACCGCTGAAGAAAGAGAATAGCACATCTAACACCAGTGATC
AGGAAATCCTCAACAAGATCCAGGAATCCCTGAAAAATGCGAAAAAGCCGATCGTGATTACAGGACACGAAA
TTATTTCATTTGGCTGGAGAAAACCGTGTCTCAGTTCATAAGCAAGACGAAACTGCCTATTACCACCTTGA
ACTTTGGTAAATCGTCCGTGGATGAAGCGTTACCGTCCTTTTTGGGGATTTACAATGGCAAACTGTCAGAGC
CGAATTTGAAGGAATTCGTGGAAAGCGCCGATTTCATTCTGATGCTTGGCGTGAAACTGACTGATAGTTCGA
CGGGTGCCTTTACTCATCACTTAAACGAGAACAAAATGATCTCTCTCAACATTGACGAGGGCAAGATTTTCA
ACGAGTCCATTCAGAACTTCGACTTCGAGTCTTTGATTAGCAGTCTGCTGGATCTGTCGGAAATCGAATACA

```
AAGGGAAATACATTGACAAGAAACAGGAAGACTTTGTACCGTCCAATGCATTACTGAGTCAAGATCGCCTGT
GGCAAGCGGTTGAGAACCTTACGCAAAGCAACGAAACCATAGTCGCCGAACAAGGTACGTCGTTTTTCGGAG
CAAGCAGCATCTTCCTGAAACCCAAATCACACTTTATCGGTCAACCACTCTGGGGTTCTATTGGTTACACCT
TTCCAGCAGCTCTCGGCTCACAGATAGCGGATAAAGAGAGTCGACACCTGCTGTTTATTGGCGATGGTTCTT
TGCAGTTGACTGTTCAGGAACTCGGCTTAGCAATTCGGGAGAAGATTAACCCCATTTGCTTCATCATCAATA
ATGACGGGTATACCGTCGAACGTGAAATCCATGGCCAAATCAAAGCTATAACGACATTCCGATGTGGAACT
ATTCGAAACTTCCGGAATCGTTTGGCGCAACTGAAGAACGTGTCGTGTCCAAAATCGTGCGTACAGAAAACG
AGTTTGTCAGCGTTATGAAAGAAGCGCAGGCAGATCCGAATCGCATGTACTGGATCGAGCTGATTCTGGCCA
AAGAAGACGCTCCGAAAGTCCTGAAGAAAATGGGCAAACTCTTCGCAGAACAGAACAAATCCTAATATAAGT
AGGAGATAAAAACATGATTAAAGCCTACGCTGCCCTGGAAGCCAACGGAAAACTCCAACCCTTTGAATACGA
CCCCGGTGCCCTGGGTGCTAATGAGGTGGAGATTGAGGTGCAGTATTGTGGGGTGTGCCACAGTGATTTGTC
CATGATTAATAACGAATGGGGCATTTCCAATTACCCCCTAGTGCCGGGTCATGAGGTGGTGGGTACTGTGGC
CGCCATGGGCGAAGGGGTGAACCATGTTGAGGTGGGGGATTTAGTGGGGCTGGGTTGGCATTCGGGCTACTG
CATGACCTGCCATAGTTGTTTATCTGGCTACCACAACCTTTGTGCCACGGCGGAATCGACCATTGTGGGCCA
CTACGGTGGCTTTGGCGATCGGGTTCGGGCCAAGGGAGTCAGCGTGGTGAAATTACCTAAAGGCATTGACCT
AGCCAGTGCCGGGCCCCTTTTCTGTGGAGGAATTACCGTTTTCAGTCCTATGGTGGAACTGAGTTTAAAGCC
CACTGCAAAAGTGGCAGTGATCGGCATTGGGGGCTTGGGCCATTTAGCGGTGCAATTTCTCCGGGCCTGGGG
CTGTGAAGTGACTGCCTTTACCTCCAGTGCCAGGAAGCAAACGGAAGTGTTGGAATTGGGCGCTCACCACAT
ACTAGATTCCACCAATCCAGAGGCGATCGCCAGTGCGGAAGGCAAATTTGACTATATTATCTCCACTGTGAA
CCTGAAGCTTGACTGGAACTTATACATCAGCACCCTGGCGCCCCAGGGACATTTCCACTTTGTTGGGGTGGT
GTTGGAGCCTTTGGATCTAAATCTTTTTCCCCTTTTGATGGGACAACGCTCCGTTTCTGCCTCCCCAGTGGG
TAGTCCCGCCACCATTGCCACCATGTTGGACTTTGCTGTGCGCCATGACATTAAACCCGTGGTGGAACAATT
TAGCTTTGATCAGATCAACGAGGCGATCGCCCATCTAGAAAGCGGCAAAGCCCATTATCGGGTAGTGCTCAG
CCATAGTAAAAATTAGTCTAGACGCAAAAAACCCCGCTTCGGCGGGGTTTTTTCGCCTGCAG
```

*The regions encoding *ccaR* (Turquoise), P_{cpcG2} (Green), Shine-Dalgarno-like sequence derived from *cpcB* from *Synechococcus* sp. PCC 7002 (Gray), *kdc* (Yellow), *adh* (Pink) and *ccaS* (Red), $P_{trc\text{-}core}$ (Dark red) were shown.

Table S5 Primer sequences used in this study.

Primer No.	Sequence (5' to 3')
**P01**	GTTTAATTACTAACTAGATCTTATAAGTAGGAGATAAAAACATGTATACGGTTGGAGATTATCTGC
**P02**	TATCTCCTACTTATATTAGGATTTGTTCTGTTCTGCGAAGAG
**P03**	CCTAATATAAGTAGGAGATAAAAACATGATTAAAGCCTACGCTGCCC
**P04**	TTACCGTCAAAAAAATCTAGACTAATTTTTACTATGGCTGAGCACTACCC
**P05**	GTTTAATTACTAACTAGATCTTATAAGTAGGAGATAAAAACATG
**P06**	CTACTTATACTAATTTTTACTATGGCTGAGCACTACCC
**P07**	GTAAAAATTAGTATAAGTAGGAGATAAAAACATGAGAATTCTTTTAGTG
**P08**	TTACCGTCAAAAAAATCTAGACTAGTTTTTCCCTTGGCACAAAG
**P09**	AATTCTGCAGGAGCTGTTGACAATTAATCATCCGGCTCGTATAATGTGTGGAAGATCTTATAAGTAGGAGATAAAAACATGTATACGG
**P10**	AATTCTGCAGGCGAAAAAACCCCGCCGAAGCGGGGTTTTTTGCGTCTAGACTAATTTTTACTATGGCTGAGCAC
**P11**	GGTTGGTAACGCCAATGAAC
**P12**	ACCAAAGGTCGTCAAGAACG
**P13**	GCTAATACCCAATGTGCCGA
**P14**	CACTGCTGCCTCCCGTAG
